# An Optical Smartphone-Based Inspection Platform for Identification of Diseased Orchids

**DOI:** 10.3390/bios11100363

**Published:** 2021-09-30

**Authors:** Kuan-Chieh Lee, Yen-Hsiang Wang, Wen-Chun Wei, Ming-Hsien Chiang, Ting-En Dai, Chung-Cheng Pan, Ting-Yuan Chen, Shi-Kai Luo, Po-Kuan Li, Ju-Kai Chen, Shien-Kuei Liaw, Choa-Feng Lin, Chin-Cheng Wu, Jen-Jie Chieh

**Affiliations:** 1 Institute of Electro-Optical Engineering, National Taiwan Normal University, Taipei 116, Taiwan; 80648003s@ntnu.edu.tw (K.-C.L.); 80648001s@ntnu.edu.tw (Y.-H.W.); 80448001s@ntnu.edu.tw (W.-C.W.); d01446001@ntu.edu.tw (M.-H.C.); 60548009s@ntnu.edu.tw (C.-C.P.); 60877006h@ntnu.edu.tw (T.-Y.C.); 60977027h@ntnu.edu.tw (S.-K.L.); 60977025h@ntnu.edu.tw (P.-K.L.); 61077015h@ntnu.edu.tw (J.-K.C.); 2 Department of Anatomy and Cell Biology, College of Medicine, National Taiwan University, Taipei 116, Taiwan; 3 Floriculture Research Center, Taiwan Agricultural Research Institute, Council of Agriculture, Yun Lin 646, Taiwan; tedai@tari.gov.tw; 4 Department of Electronic and Computer Engineering, National Taiwan University of Science and Technology, Taipei 106, Taiwan; skliaw@mail.ntust.edu.tw; 5 Department of Electronic Engineering, Oriental Institute of Technology, New Taipei 220, Taiwan; ff019@mail.oit.edu.tw; 6 Chemical Systems Research Division, National Chung-Shan Institute of Science & Technology, Taoyuan 325, Taiwan; albert0830.eed99g@nctu.edu.tw

**Keywords:** optical inspection, diseased orchids, artificial intelligence, Internet of Things

## Abstract

Infections of orchids by the *Odontoglossum ringspot virus* or *Cymbidium mosaic virus* cause orchid disfiguration and are a substantial source of economic loss for orchid farms. Although immunoassays can identify these infections, immunoassays are expensive, time consuming, and labor consuming and limited to sampling-based testing methods. This study proposes a noncontact inspection platform that uses a spectrometer and Android smartphone. When orchid leaves are illuminated with a handheld optical probe, the Android app based on the Internet of Things and artificial intelligence can display the measured florescence spectrum and determine the infection status within 3 s by using an algorithm hosted on a remote server. The algorithm was trained on optical data and the results of polymerase chain reaction assays. The testing accuracy of the algorithm was 89%. The area under the receiver operating characteristic curve was 91%; thus, the platform with the algorithm was accurate and convenient for infection screening in orchids.

## 1. Introduction

Orchid cultivation is a crucial industry globally because of the wide use of ornamental plants for festivals or commercial purposes. However, in large-scale orchid farms or cargo, infection with *Odontoglossum ringspot virus* (ORSV) or *Cymbidium mosaic virus* (CyMV) [1] can disfigure orchids, resulting in large financial losses or export embargoes. Current inspection techniques for incubating infections include observation of appearance based on personal experience and biological assays that are costly and require both time and labor. Commonly used test-strip immunoassays [2,3] and enzyme-linked immunosorbent assays [4] have poor performance due to their brief effective timeframes, in addition to susceptibility to operator error and limited sensitivity.

Recent immunoassay developments such as immunomagnetic reduction using magnetic nanoparticles as virus labelers [5] and biochips enabling high-resolution imaging [6,7] have increased sensitivity by several parts per billion (ppb). The gold standard—polymerase chain reaction (PCR)—has the highest sensitivity but is also the costliest [8]. However, both conventional and newer biological assays are suitable for only sampling inspection but not for general screening. Sampling inspection cannot guarantee the absence of infection in unexamined pots and may damage the appearance of the plants in examined pots, as well as increasing the infection risk.

General screening requires strict inspection methods with high performance that are nondestructive, rapid, and inexpensive in terms of time, money, and labor. For example, the analysis of the bioconditions of target materials uses the intrinsic autofluorescence characteristics of organic materials, as opposed to the addition of artificial fluorescence markers to biopsies [9]. However, fluorescence microscopy requires a pure protein extraction from a biopsy [10]. Laser-induced fluorescence technology involves exciting certain atoms, molecules, or organic objects with a single-wavelength light source, such as a laser or light-emitting diode (LED), to analyze the fluorescence [11] of complex samples. However, a measurement technology must be sensitive enough to capture weak fluorescence and to discriminate between excitation and fluorescence. Statistics and algorithms can be used to perform an analysis of species’ target parameters. For example, laser-induced chlorophyll fluorescence (LICF) has been used to characterize chlorophyll and thus investigate plant growth, water deficits, ambient light, and temperature [12,13,14,15]. Researchers have discovered critical findings regarding LICF: green light is superior to ultraviolet (UV) light for excitation [15], and for algorithmic or analytic methods, the intensity ratio of red and infrared lights around 690 and 730 nm affects the biological response [12,13,14]. Other parameters and methods using commercial statistical software have also been investigated in the literature [15].

However, these algorithmic methods remain limited to well-controlled experiments with significant signals; therefore, artificial intelligence (AI)-based methods have been validated for complex applications [16,17,18]. As the Extreme Gradient Boosting algorithm (XGBoost) uses the decision tree of AI learning algorithm and includes a second-order Taylor expansion on the loss function for increased accuracy and loss function customization [16], XGBoost is more suitable for data analysis than other AI algorithmic methods. For example, kernel-based methods for projecting data into different dimensions, such as support vector machines [17], only include a first-order Taylor expansion with a gradient boosting decision tree as the loss function [18].

In addition, powerful LICF instruments are often heavy, bulky, and expensive, and AI algorithms require large databases and high-performance computers to achieve satisfactory performance. Therefore, this study proposed an AI and Internet of Things (AIoT)–based platform for detecting orchid infection [19,20]. An inexpensive and handheld inspection tool was fabricated for this study and connected to an Android smartphone. A cloud-based database and AI algorithm were developed, and an Android app was coded to display the results of the measurement and algorithm.

## 2. Materials and Methods

### 2.1. Biological Samples and Protocol

**Figure 1:**
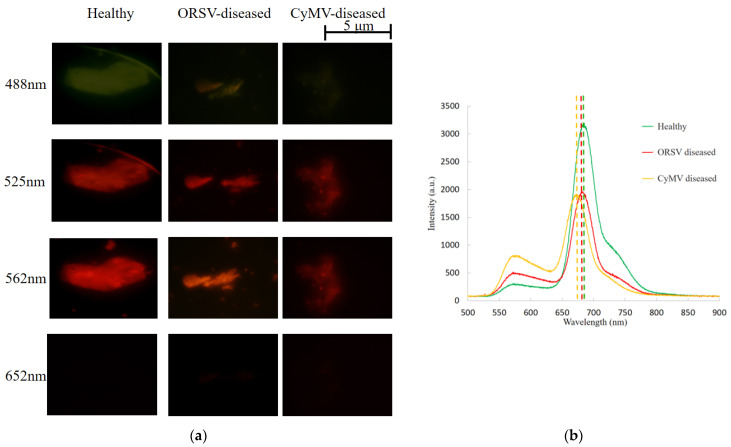
Optical fluorescence characterization of the extracted solutions of *Odontoglossum ringspot virus* (ORSV)- or *Cymbidium mosaic virus* (CyMV)-diseased, ORSV-diseased, and healthy plants. (**a**) Fluorescence microscopy images under 652, 562, 525, and 488 nm excitation light. (**b**) Fluorescence spectra under 532 nm green laser excitation as displayed by the optical spectrum analyzer (OSA).

**Figure 2:**
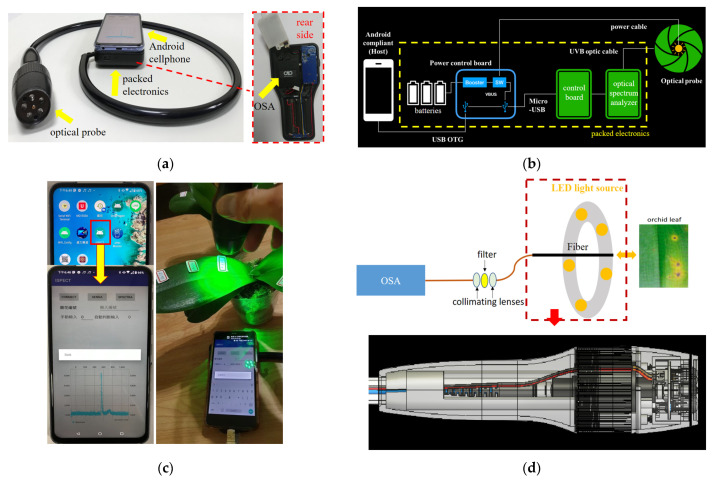
Handheld artificial intelligence (AI) and Internet of Things–based inspection platform. (**a**) Photograph of the system. (**b**) Schematic of the system. (**c**) Icon and main panel of the app, displaying the leaf spectrum under green-light excitation, as measured by the optical probe. (**d**) Schematic of the optical probe and components of the inspection platform.

The fluorescence of 0.1 mL chlorophyll solutions extracted from healthy plants, plants with CyMV, and plants with ORSV (Dr. Chip Biotechnology Inc., Miaoli Country, Taiwan) was investigated under excitation light with wavelengths of 488, 525, 562, and 652 nm under a fluorescence microscope (IX-71, Olympus Corporation, Tokyo, Japan; Figure 1a). To quantitate the difference in fluorescence among the extracted chlorophyll solutions under 532 nm green laser excitation (Figure 1b), their fluorescence spectra were examined using an optical spectrum analyzer (OSA; ultra-micro spectrometer module AM2280, OTO Photonics, Hsinchu City, Taiwan). The OSA was 78 mm × 153 mm × 24 mm (right chart in Figure 2a), and its power consumption of 4.5 V and 350 mA was supported by three replaceable AA alkaline batteries (Figure 2b).

**Figure 3:**
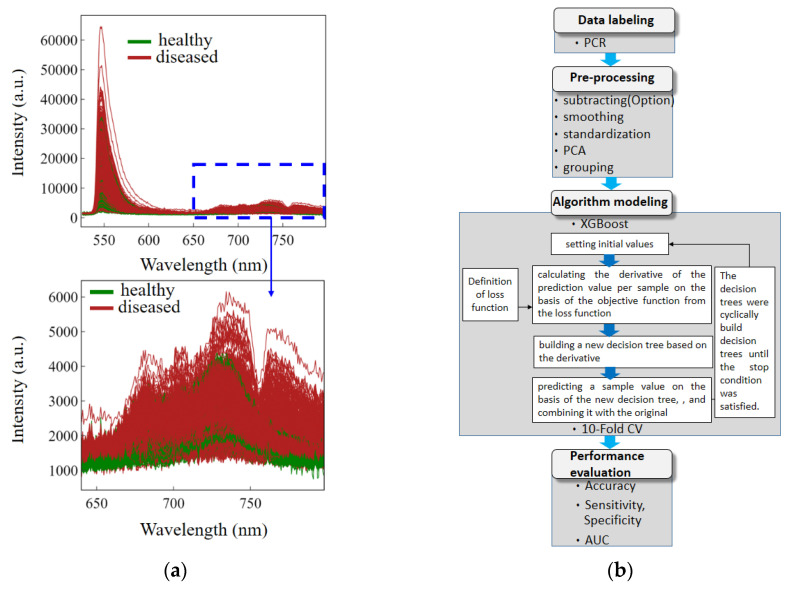
Raw data and AI algorithmic processing of optical records. (**a**) Measured optical spectra, *n* = 3643. (**b**) Algorithmic modeling and model evaluation.

The orchid incubation protocol lasted 2 months, and the healthy and diseased orchids were optically measured every week in vivo (Figure 2c and Figure S1). A PCR measurement of the biopsy samples was performed at the end of the process. The 24 healthy orchids, all of the same age, were divided equally into two groups for separate incubation. In one group, the dilute chlorophyll extract solutions from the CyMV or ORSV plants were applied to their pot soil and leaves to incubate the disease. During the 2-month incubation, two leaves per orchid pot and three measurement points per leaf were measured weekly using the handheld AIoT platform, generating 3634 optical measurements (Figure 3a). Each orchid usually had only one leaf with sufficient area for optical tests of three different points. The measurement distance between the leaf and the optical probe was approximately 1 cm to control the illuminated region.

The 144 measurement points were then cut and used for PCR measurement (Dr. Chip Biotechnology In., Miaoli Country, Taiwan; Table S1). According to the PCR results, 30 and 114 samples from 5 and 19 orchid pots were identified as diseased and healthy, respectively.

### 2.2. Handheld AIoT-Based Platform and App

We developed a handheld AIoT-based platform consisting of an optical probe, an OSA, electronics, and an Android smartphone (Figure 2a,b). The optical probe consisted of two parts (Figure 2a,d). The first part was the excitation light source: five LEDs (525–532 nm, Lite-On LTL2R3TGY3KS-032A, Lite-On Technology Corp., Taipei, Taiwan) arranged in rings for uniform illumination and powered by 3 V from a circuit board. The second part was the optical collector at the center of the rings. An optical fiber (OF-600-100-UVB, OTO Photonics, Hsinchu City, Taiwan) with a small numerical aperture (NA) of 0.22 was used. The NA of a fiber is the sine of the maximal input angle of an incident ray that allows for the propagation in the fiber core through total internal reflection. A small-NA fiber was used as a receiver to filter out the background light, i.e., rays of randomly incident angles, leaving only ultra-few optical rays with the smaller incident angles than the NA-based incident angle [21]. In front of the OSA, two collimating lenses (COL-OF-S, OTO Photonics, Hsinchu City, Taiwan) were placed in front of and behind the 550 nm optical longpass filter (#62-983, Edmund Optics Ltd., York, UK; Figure 2c). Finally, 330–850 nm light was detected by the OSA (UM2280, OTO Photonics, Hsinchu City, Taiwan). The OSA was mounted on a control board (CB-56M2, OTO Photonics, Hsinchu City, Taiwan) with a universal serial bus interface to output the spectral data (Figure 2a). The optical probe and OSA used three AA batteries and were modulated by a power control module. The data from the OSA were output to the Android smartphone. The electronics and optical probe were protected with thick, elastic plastic shells.

The Android app was connected to the Internet and could display the measured spectrum and the results of algorithmic detection (i.e., positive or negative). The app uploaded the data descriptions and measured spectra to the cloud server (Figure 2d). The cloud server used an i7-6700 central processing unit at 3.4 GHz and a 71 Mbps Internet connection.

A characterization test was performed to examine background suppression and the influence of leaf thickness. Three samples from a single orchid leaf approximately 3 mm thick, a rough piece of paper with a similar thickness for reference, and double cascade of leaves approximately 6 mm thick were measured at a fixed distance of approximately 1 cm from the optical probe in indoor and outdoor settings. The background spectrum was automatically measured for comparison with the database level as a self-check procedure when the app was launched. If the background spectrum was 5 times higher than the criterion level, it was temporally selected as the reference for subtraction in spectral measurements until the app was closed or restarted for another self-check. This optional subtraction of the initial high background was the first step of the app spectrum measurement (Figure 3b).

### 2.3. AI Algorithm for Processing Optical Records

The AI algorithm processed the 3634 optical records (Figure 3a) in several steps (Figure 3b). The PCR data labeling revealed 715 healthy and 2919 diseased data points, a ratio of 1:4. The preprocessing steps were designed to suppress interference through smoothing and standardization and then to collect features through principal component analysis (PCA) and grouping. The smoothing and standardization algorithms were based on Savitzky–Golay smoothing [22] and standard deviation normalization, respectively [23] (Figure S2). For the standardization, only 640–800 nm fluorescence was analyzed through PCA (Figure 3a) [24]. The fluorescence intensity for each wavelength point was used as a dimension, for a total of 238 dimensions. A total of 11 PCAs were selected from the 238 dimensions because this combination yielded the highest average of training and testing scores among various numbers of PCA pairs (Table S2). The 3634 data points were divided into a training group, consisting of 2543 data points, and a testing group, consisting of 1091 data points. The ratio of data points in the training group to those in the testing group was 7:3, and the ratio of diseased to healthy samples was 1:4 in both groups.

The PCAs were input into XGBoost, which was used to cyclically build decision trees until the stop condition was satisfied (Figure 3b). The process involved setting initial values, calculating the derivative of the prediction value per sample on the basis of the objective function from the loss function, building a new decision tree based on the derivative, predicting a sample value on the basis of the new decision tree, and combining it with the original. The parameters (Figure S60) were optimized through a grid search tuning technique, and the decision trees (Figure S61) were structured after the algorithm cycles had concluded. Subsequently, a 10-fold cross-validation (CV) method was used with 10 training subgroups for self-testing. Each of the subgroups comprised 10% of the training data and contained data from diseased and healthy plants in a ratio of 1:4. The high average accuracy of 0.856 across the 10 training subgroups indicated that the model was reliable (Table S2).

## 3. Results

### 3.1. Fluorescence Wavelength Variation with Disease Status

The fluorescence of some of the extracted chlorophyll in approximately 5–10 µm chloroplasts was observed through fluorescence microscopy at 500× magnification with 652, 562, 525, and 488 nm excitation sources (Figure 1a). No fluorescence was observed in the chlorophyll excited by 652 nm red light, but weak green fluorescence was observed under 488 nm blue light. Red fluorescence was observed under 525 and 562 nm green light, but the difference among three biological conditions of health, CyMV-disease, and ORSV-disease was difficult to distinguish. Therefore, a 532 nm green laser was used to excite these extracted chlorophyll solutions, and the optical spectra revealed small differences between the peak wavelengths of healthy and diseased plants (Figure 1b). The fluorescence peaks were at 669, 684, and 691 nm for the CyMV-diseased, ORSV-diseased, and healthy plants, respectively.

### 3.2. Handheld AIoT-Based Inspection Platform

The handheld AIoT-based platform exhibited satisfactory performance in some aspects of optical measurement and signal transmission. For example, the small-NA optical fiber in the probe measured the fluorescence and suppressed environmental light and the excitation light emitted by the LEDs. The two collimating lenses modulated the light emitted by the optical fiber and transmitted it to the filter, after which it entered the OSA (Figure 2d). This greatly reduced the intensity of the excitation light reflected by the plant leaves, thus preventing the OSA from being saturated with excitation light. In the characterization test, the spectra of the rough piece of paper, single orchid leaf, and double cascade leaves in indoor and outdoor settings were analyzed in the green (530–600 nm) and red fluorescence bands (640–800 nm). In the green band, differences associated with the illuminated materials, numbers of leaves, and settings were observed. However, in the red band, no differences in profile or intensity were associated with the numbers of leaves or the setting. Only a difference associated with the illuminated material was observed; the paper did not fluoresce, whereas the leaves did (Figure S3).

The entire procedure, which required 1000 ms for the *token*-based connection to the server (once every 2 h), 200 ms for measurement, uploading, and downloading, 500 ms for server calculation, and 100 ms for result display, required less than 3 s (Figure 2c). The processing of the AI algorithm by using the server rather than the smartphone app was not only faster but also ensured the algorithm remained unknown and that it could be improved. For example, the app could be used at orchid farms worldwide to upload the results of plant inspection, and the specificity and precision of the algorithm could be increased.

### 3.3. AI Algorithm and Performance Evaluation for Nondestructive Measurement

The optical measurement focused on weak red fluorescence (640–800 nm), indicated by a dashed rectangle in the upper part of Figure 3a, rather than on the large amount of reflected green excitation light emitted by the LEDs (525–532 nm), represented by the peak in the chart on the upper-left section of Figure 3a. However, unlike the clearly observable fluorescence peak variation of the chlorophyll solutions extracted from the healthy and diseased plants (Figure 1b), the fluorescence spectra of the leaves were too complex to distinguish visually (Figure 3a) because of the greater variety of materials in the plants than in the extracted chlorophyll solutions. Therefore, the AI algorithmic preprocessing stage was used to remove the signal from the noise and non-chlorophyll-based materials.

The entire procedure of this study comprised data labeling, preprocessing, algorithmic modeling, and model evaluation (Figure 3b). First, the 715 and 2919 optical data points from 30 diseased and 114 healthy samples, respectively, were labeled after real-time PCR over the course of 2 months (Table S1). Fewer data points were labeled diseased than healthy because the infection rate was lowered through the application of diluted chlorophyll extract solutions from the CyMV and ORSV plants to the soil and leaves during the 2-month incubation. In Figure 3a, the high left peak is the excitation light, whereas the low and wide plateau on the right represent the fluorescence. The fluorescence band is shown in detail in the lower part of Figure 3a.

The second step was data preprocessing, including smoothing, standardization, PCA, and grouping (Figure 3b). In the smoothing step, noise was filtered from the spectra (Figure S2a) by using Savitzky–Golay smoothing. The resulting spectral profiles were then standardized by subtracting the mean and then normalized by dividing by its standard deviation (Figure S2b) to suppress noise and the effects of measurement distance. In the third preprocessing step of the PCA method, the 238 intensity datapoints for the fluorescence band between 640 and 800 nm in the smoothed and normalized spectrum were used as the input data to create 238 dimensions. Decreasing the number of preceding-order PCAs with large eigenvalues decreased pair variance substantially but increased training scores, testing scores, and the testing area under the receiver operating characteristic (ROC) curve (AUC) until they stabilized at six PCAs (Table S2). The cutoff was the point at which the average of the training and testing scores was highest (0.868): 11 PCAs with large eigenvalues.

**Figure 4:**
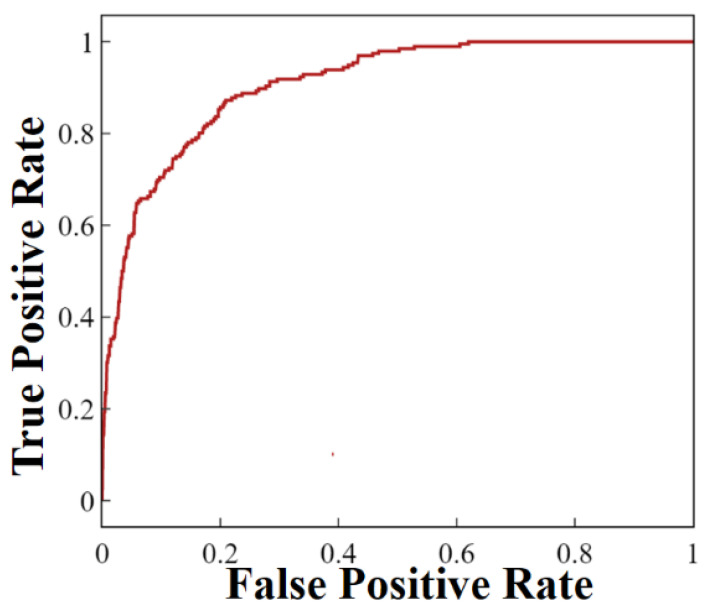
Receiver operating characteristic curve of the algorithmic model for the testing group (*n* = 1091).

In the final preprocessing step (grouping), the 3634 records were randomly divided into a training group of 2543 records (70%) and a testing group of 1091 records (30%). The training group was used for further algorithmic modeling using the XGBoost method. The 11 PCAs of each optical record were used in accordance with the method described in the algorithmic modeling flowchart (Figure S58). The decision tree (Figure S59) was determined to be the appropriate algorithm and was validated through 10-fold CV (Table S3) [25]. The 10 test scores from the 10-fold CV represented the accuracies of each of the 10 training subgroups, and the average value, i.e., the training accuracy (based on PCR labeling), was approximately 0.856. Correct and incorrect predictions of PCR labeling were labeled as true (T) or false (F), respectively. Diseased and healthy plants were labeled as positive (P) or negative (N) according to the PCR results. Each data point was then labeled as true negative (TN), true positive (TP), false negative (FN), or false positive (FP) in a confusion matrix (Table 1). The correct predictions (gray, Table 1) were 859 TNs and 111 as TPs. The numbers of FNs and FPs were 85 and 36, respectively. The accuracy, defined as the proportion of correct predictions [(TN + TP)/(TN + FP + TP + FN)], was 0.889. The specificity of the prediction [TN/(TN + FP)] was 0.960, and the sensitivity [TP/(TP + FN)] was 0.566. However, the precision [TP/(TP + FP)] was approximately 0.755, and the negative predictive value [TN/(TN + FN)] was approximately 0.91. The training accuracy based on the 10-fold CV of 0.856 was close to the testing accuracy of 0.889. Furthermore, the ROC curve was plotted for general classification algorithms. Through reduction of the criteria for positive or negative discrimination, the TP rate (sensitivity) increased rapidly with the FP rate to perfect sensitivity (FP rate of approximately 0.64; Figure 4). Hence, the AUC was 0.91, close to a perfect value of 1, for a square-profile ROC curve.

## 4. Discussion

The qualitative analysis revealed green and red fluorescence under short excitation wavelengths of 488, 525, and 562 nm but not under 652 nm light. This can be explained by the principle of fluorescence: light with higher photon energy, such as blue and green light, can excite leaves to fluoresce with long wavelengths, but light with low photon energy, such as red light, cannot (Figure 1a). Among the three types of fluorescence exhibited under the same excitation intensity, the green fluorescence produced by the blue excitation light was weaker than the red fluorescence produced by the green excitation light because some parts of the green fluorescence were absorbed again, but were too weak to induce the observable red fluorescence. This result is consistent with findings regarding LICF and the use of green-light lasers for excitation [12,13,14,15]; ultimately, green excitation light was selected for this study. The chlorophyll solutions extracted from the healthy plants exhibited stronger fluorescence under 488, 525, and 562 nm light than did those extracted from the diseased plants (Figure 1a). This was quantitatively verified: the fluorescence intensity of the chlorophyll extracted from the healthy plants was higher than that of the chlorophyll extracted from the diseased plants under 532 nm green-light laser excitation as well as in the green band between 525 and 562 nm (Figure 1b). In addition, the fluorescence peaks of the chlorophyll solutions extracted from ORSV- and CyMV-diseased plants were shifted to wavelengths 10 and 20 nm lower than those of the solutions from healthy plants (Figure 1b). The left-shift of the fluorescence peaks in extract solutions is consistent with the dark flecks or yellowing symptoms observed in diseased orchid plants and with the lower wavelength of the fluorescence peak of brown-colored fucoxanthin-chlorophyll a/c-binding proteins than the fluorescence peaks of chlorophyll a and c [26]. This difference in the fluorescence of the chlorophyll a and c from the diseased plants (Figure 1b) is consistent with the decrease in the fluorescence intensity of the chlorophyll from the healthy plants (Figure 1a,b).

Furthermore, the small difference in the fluorescence peak wavelengths between the healthy and diseased extraction solutions was not discernible in photos (Figure 1a). Although CyMV- and ORSV-diseased orchids could be easily differentiated from healthy orchids by using the extracted solutions (Figure 1b), destructive sampling using biopsies has higher time and labor costs and presents a risk of infection. Therefore, standard LICF based on the ratio of the two chlorophyll fluorescence maxima can be used to nondestructively inspect leaves for large variations in simple biological factors, such as sun illumination [13,14,26]. Unlike the high-level optical devices used for standard LICF (expensive green-light lasers and heavy systems), the compact system (the green LEDs and handheld AIoT platform) developed in this study enables early detection of infected chlorophyll through the PCA and XGBoost components of the AI algorithm. The proposed handheld AIoT platform is superior to LICF and boasts a unique optical measurement system and algorithm.

Distinguishing healthy from diseased chlorophyll solutions under green light is possible, but doing so through nondestructive means is difficult because of the interference from background noise, the presence of organic materials in leaves, and parameters other than those affected by CyMV or ORSV. The small-NA fiber can eliminate background light outside a small input angle range. Only slight intensity differences between indoor and outdoor background were exhibited in the red fluorescence band (Figure S3). In addition, no difference in the fluorescence profile or intensity was observed between the samples with one and two leaves (Figure S3). For any thick or non-uniform-distribution samples, coverage materials or different materials influenced the fluorescence from deeper organic materials through wavelength-dependent scattering [14] or the selective reabsorption of red fluorescence [27]. The scatter effect widens the angle of the fluorescence rays emitted by a leaf, whereas reabsorption reduces fluorescence intensity. Hence, for thick samples, the evaluation indicatory F690/F730 of LICF measurement became lower than thin ones, but the small-NA fiber can suppress the scattered fluorescence of deeper chlorophyll. Figure S3 indicates that the fluorescence detected was different because of the intense reflection of the excitation light, which was influenced by several complex parameters more than by differences in ray angles, surface roughness, and thickness of the sample materials.

In the algorithm, when few PCAs were used, such as only two pairs of the 11 PCAs, the ROC curves (Figures S4–S58) and their AUC values (Figure S59) varied considerably. Qualitatively, the pairs of preceding-order PCA with larger eigenvalues resulted in superior performance. For example, the top two AUC values corresponded to the pairings of the second and fourth PCAs and the first and second PCAs. Similar to the evaluation indicator of F690/F730 used for LICF [14,27], only two intensities of two wavelengths were insufficient to suppress the complex influences of various factors. However, although more than six PCAs were selected for pairing, only PCAs with small eigenvalues were added and yielded the gradual saturation in several results, such as the stabilization of the training score, testing score, and testing AUC (Table S2). While 11 PCAs were selected, the average of the training and testing scores was achieved for the maximal value. Further, the TP, TN, FP, and FN values (Table S4) as well as the AUC, sensitivity, and specificity differed limitedly among the pairs of 10–12 PCAs. For example, the optimal specificity of 0.96 was achieved with 11 PCAs; specificity was 0.93 and 0.94 with 10 and 12 components, respectively. Sensitivity increased from 0.566 with 11 components to 0.628 and 0.622 with 10 and 12 components, respectively. However, the AUC values for 10–12 components were similar, approximately 0.91. The average accuracy of approximately 0.856 yielded by testing with 10-fold CV (Table S3) validated the decision tree algorithm of the XGBoost model. Consequently, the accuracy based on the elements of the confusion matrix (Table 1) and the AUC of the testing group results (Figure 4) were 0.889 and 0.91. In other words, the correct prediction rates for both the positive and negative conditions were excellent. The specificity and negative predictive value of approximately 0.960 and 0.91, respectively, were also excellent. However, the sensitivity and precision of 0.566 and 0.755, respectively, were both substantially lower than the overall accuracy, and resulted from low TPs and high FNs (Table 1). Although the TP rate was always higher than the FP rate, as demonstrated by the ROC curve (Figure 4), the TPs were still few as one reason of the low sensitivity and the low precision. The overall low infection rate in this work yielded few diseased results, i.e., low TPs and low FNs. The high FNs resulted from the current protocol that optical data were measured every week, but PCR sampling was performed only at the end of the second month; therefore, all diseased plants were measured multiple times before they finally became infected. Consequently, labeling negative optical data as the wrong positive records induced the FN results in algorithm. Moreover, both high specificity and non-high precision came from low FPs (Table 1). Here, in addition to the error algorithm, FPs may occur from similar light-yellow coloration in leaves with diseased ones due to metabolic stress, biological stress, or other factors. Overall, the phenomenon of high specificity and low sensitivity resulted from the algorithm learning based on the unbalanced ratio of 715 records for diseased plants to 2919 records for healthy plants (1:4), in the range of 1:4 to 1:100 [28]. Two methods could be adopted to increase the sensitivity. The immediate solution is to adjust the number of PCAs (Table S4); however, the increase in sensitivity is limited, increasing to only 0.63 in this study. The other solution is to employ a protocol with higher costs for the inspection of diseased and healthy plants at a lower ratio than 1:4. Here, varied and numerous diseased plants could increase the TPs. Furthermore, the optical measurements of each plant should be conducted only soon before sampling the PCR biopsy to decrease the wrong positive records for the generation of FNs. In the inspection protocol, these modifications of TP increase and the FN decreases had great potential to promote the sensitivity.

With the mentioned rapid measurement speed within 3 s and high specificity of 0.96, this proposed inspection could achieve not only the numerous and wide distribution of plants in indoor farms within few hours, but also the retention of healthy (TN) plants in farms without any risks, as well as the exclusion of abnormal plants (i.e., non-TN for infection), including diseased (TP and FN) plants and yellowing plants (one part of FP plants), indicating inferior growth conditions. The regular health examinations screened out the abnormal patients based on the high specificity of healthy conditions, and then subsequent high-level inspection instruments or treatment methods should be used based on the sensitivity of individual diseases. In contrast, most farmers always just discard abnormal plants without the further inspection or treatment because of the low cost of agricultural products.

## 5. Conclusions

In summary, a screening method for orchids at risk of CyMV or ORSV diseases was developed and based on variations in chlorophyll. The handheld, smartphone-based device is convenient for general screening and can be used to accumulate big data for techniques that can improve the algorithm’s performance.

## Figures and Tables

**Table 1:** Confusion matrix. Polymerase chain reaction labeling and optical prediction results for the testing group (*n* = 1091).

	PCR	Diseased (Positive)	Healthy (Negative)
Prediction			
Diseased (Positive)		111 (TP)	36 (FP)
Healthy (Negative)		85 (FN)	859 (TN)

## Data Availability

The data presented in this study are available in supplementary material here.

## Supplementary Materials

The following are available online at https://www.mdpi.com/article/10.3390/bios11100363/s1, Figure S1: Photos of the diseased and healthy groups, Figure S2: Preprocessing of the measured optical spectrum. (a) Smoothing; (b) normalization. *n* = 2, Figure S3: The spectrum of single leaf and double leaves in the indoor and outdoor environment, Figure S4: The ROC curve of 1st and 2nd PCA components, Figure S5: The ROC curve of 1st and 3rd PCA components, Figure S6: The ROC curve of 1st and 4th PCA components, Figure S7: The ROC curve of 1st and 5th PCA components, Figure S8: The ROC curve of 1st and 6th PCA components, Figure S9: The ROC curve of 1st and 7th PCA components, Figure S10: The ROC curve of 1st and 8th PCA components, Figure S11: T The ROC curve of 1st and 9th PCA components, Figure S12: The ROC curve of 1st and 10th PCA components, Figure S13: The ROC curve of 1th and 11th PCA components, Figure S14: The ROC curve of 2nd and 3rd PCA components, Figure S15: The ROC curve of 2nd and 4th PCA components, Figure S16: The ROC curve of 2nd and 5th PCA components, Figure S17: The ROC curve of 2nd and 6th PCA components, Figure S18: The ROC curve of 2nd and 7th PCA components, Figure S19: The ROC curve of 2nd and 8th PCA components, Figure S20: The ROC curve of 2nd and 9th PCA components, Figure S21: The ROC curve of 2nd and 10th PCA components, Figure S22: The ROC curve of 2th and 11th PCA components, Figure S23: The ROC curve of 3rd and 4th PCA components, Figure S24: The ROC curve of 3rd and 5th PCA components, Figure S25: The ROC curve of 3rd and 6th PCA components, Figure S26: The ROC curve of 3rd and 7th PCA components, Figure S27: The ROC curve of 3rd and 8th PCA components, Figure S28: The ROC curve of 3rd and 9th PCA components, Figure S29: The ROC curve of 3rd and 10th PCA components, Figure S30: The ROC curve of 3rd and 11th PCA components, Figure S31: The ROC curve of 4th and 5th PCA components, Figure S32: The ROC curve of 4th and 6th PCA components, Figure S33: The ROC curve of 4th and 7th PCA components, Figure S34: The ROC curve of 4th and 8th PCA components, Figure S35: The ROC curve of 4th and 9th PCA components, Figure S36: The ROC curve of 4th and 10th PCA components, Figure S37: The ROC curve of 4th and 11th PCA components, Figure S38: The ROC curve of 5th and 6th PCA components, Figure S39: The ROC curve of 5th and 7th PCA components, Figure S40: The ROC curve of 5th and 8th PCA components, Figure S41: The ROC curve of 5th and 9th PCA components, Figure S42: The ROC curve of 5th and 10th PCA components, Figure S43: The ROC curve of 5th and 11th PCA components, Figure S44: The ROC curve of 6th and 7th PCA components, Figure S45: The ROC curve of 6th and 8th PCA components, Figure S46: The ROC curve of 6th and 9th PCA components, Figure S47: The ROC curve of 6th and 10th PCA components, Figure S48: The ROC curve of 6th and 11th PCA components, Figure S49: The relationship distribution of 7th and 8th PCA components, Figure S50: The ROC curve of 7th and 9th PCA components, Figure S51: The ROC curve of 7th and 10th PCA components, Figure S52: The ROC curve of 7th and 11th PCA components, Figure S53: The ROC curve of 8th and 9th PCA components, Figure S54: The ROC curve of 8th and 10th PCA components, Figure S55: The ROC curve of 8th and 11th PCA components, Figure S56: The ROC curve of 9th and 10th PCA components, Figure S57: The ROC curve of 9th and 11th PCA components, Figure S58: The ROC curve of 10th and 11th PCA components, S59: The AUC of ROC curves varied with two PCA component pairs among chosen principal components, Figure S60. The flow chart and parameters in the algorithm modeling using XGBoost, Figure S61. The decision trees in the algorithm modeling using XGBoost, Table S1: PCR results of cut leaf points, Table S2: The pair formation of different PCA components amount, Table S3: Validation of developed algorithm model by 10-fold CV, Table S4. The performance of the top 3 pairs.
