# Supplementary material for: An Optical Smartphone-Based Inspection Platform for Identification of Diseased Orchids
Source: Biosensors (Basel). 2021 Sep 30;11(10):363. doi: 10.3390/bios11100363 (PMC8533836; doi:10.3390/bios11100363)
Supplement: Supplementary file 1 [file biosensors-11-00363-s001.zip › biosensors-1334050-supplementary.pdf]

Supplementary materials

Table S1. PCR results of cut leaf points

Label		Amount
**Negative**		114
**Positive**	**CymV**	24
	**ORSV**	6
**Total**		144

Table S2. The pair formation of different PCA components amount.

Amount of PCA Components	Training score	Testing score	Testing AUC	Variance (Eigenvalues)	Average of Training Score and Testing Score
1	0.669	0.680	0.613	6.165	0.675
2	0.768	0.816	0.778	1.558	0.792
3	0.800	0.838	0.836	1.015	0.819
4	0.809	0.849	0.872	0.238	0.829
5	0.833	0.874	0.901	0.175	0.854
6	0.847	0.878	0.902	0.103	0.863
7	0.842	0.880	0.907	0.051	0.861
8	0.844	0.874	0.897	0.049	0.859
9	0.841	0.882	0.904	0.042	0.862
10	0.849	0.875	0.898	0.037	0.862
11	0.856	0.879	0.902	0.031	0.868
12	0.854	0.877	0.903	0.028	0.866
13	0.849	0.880	0.908	0.026	0.865
14	0.845	0.873	0.908	0.021	0.859

Table S3. Validation of developed algorithm model by 10-fold CV.

Self-testing Time	1	2	3	4	5	6	7	8	9	10	Average
**Test score**	0.839	0.843	0.859	0.850	0.870	0.846	0.858	0.878	0.839	0.874	0.856

Table S4. The performance of top 3 pairs.

amount of PCA components	TN	FN	FP	TP	specificity	sensitivity	AUC
10	832	73	63	123	0.930	0.628	0.90
11	859	85	36	111	0.960	0.566	0.91
12	842	74	53	122	0.940	0.622	0.91

Diseased orchid group **Healthy orchid group**

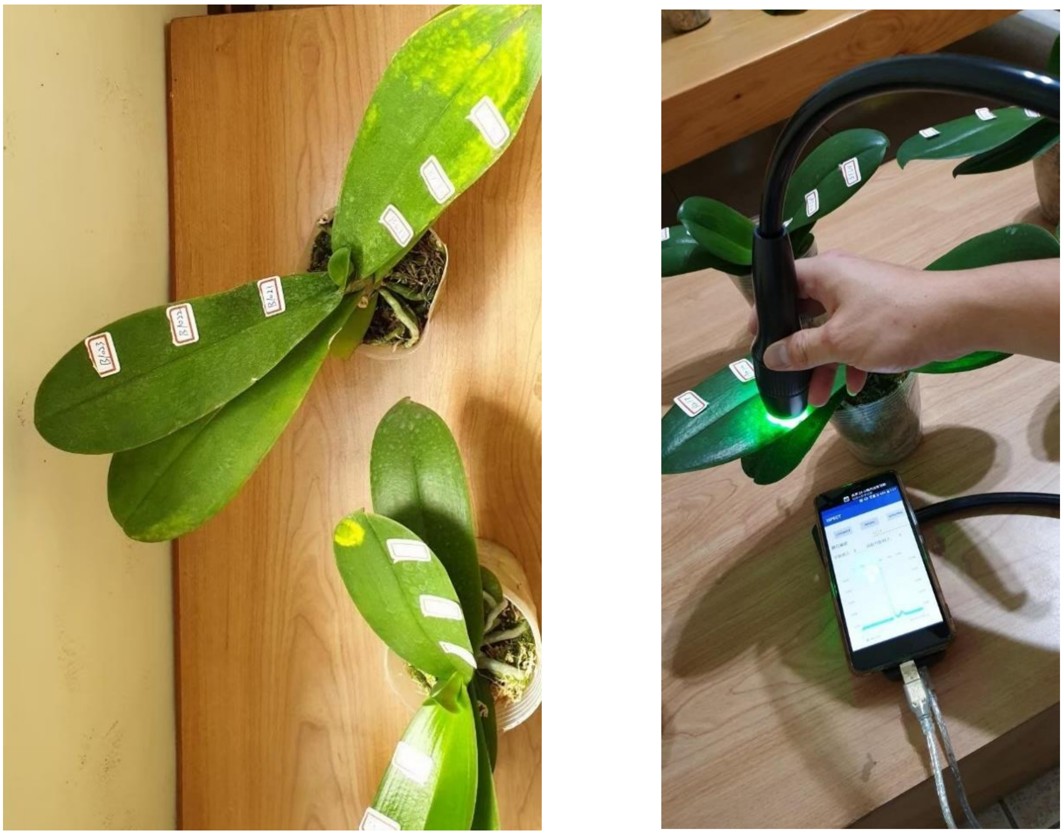

Figure S1. The photos for the diseased and healthy groups

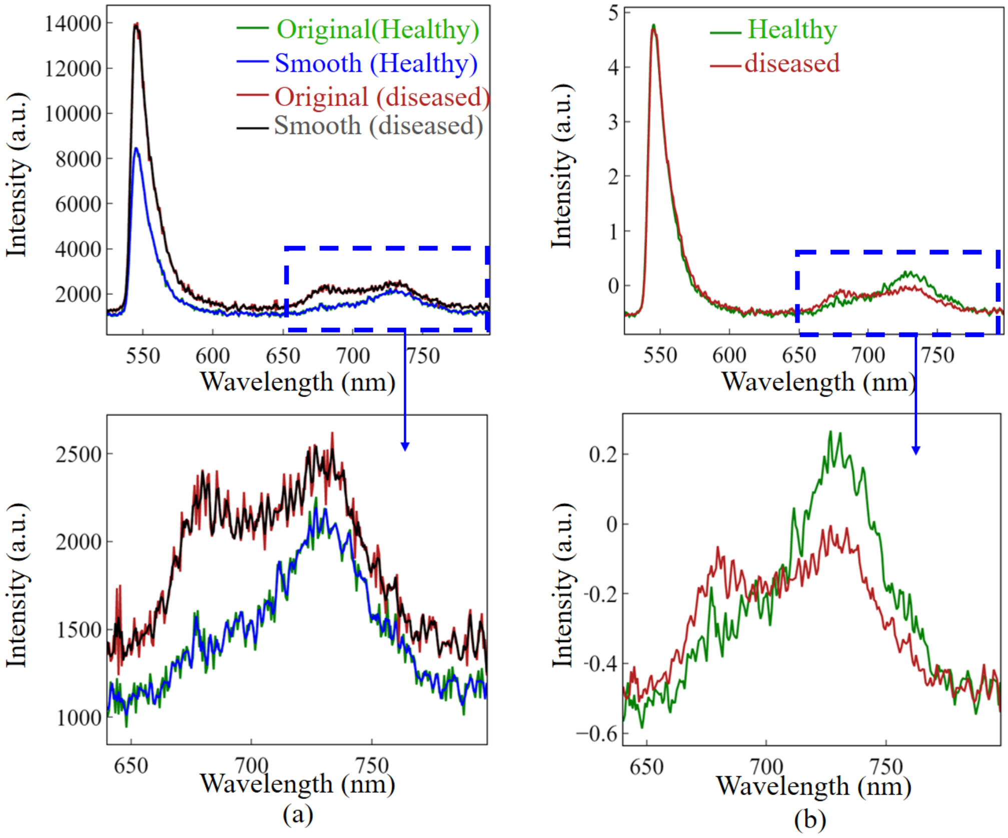

Figure S2. Preprocessing of the measured optical spectrum. (**a**) Smoothing; (**b**) normalization. n = 2.

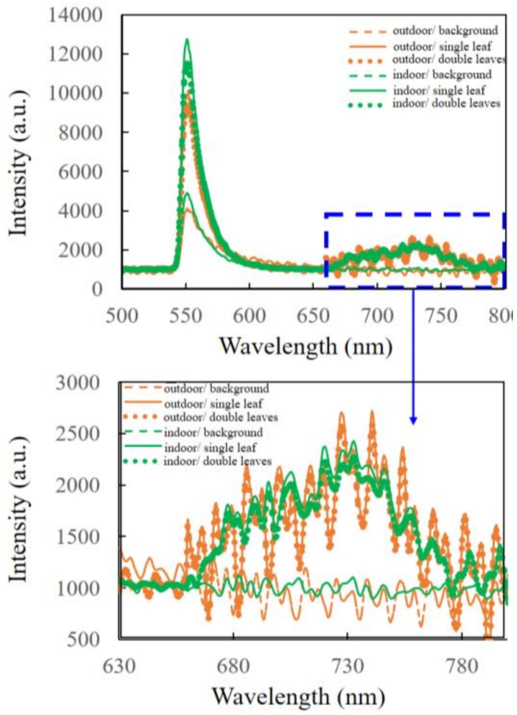

Figure S3. The spectrum of single leaf and double leaves in the indoor and outdoor environment.

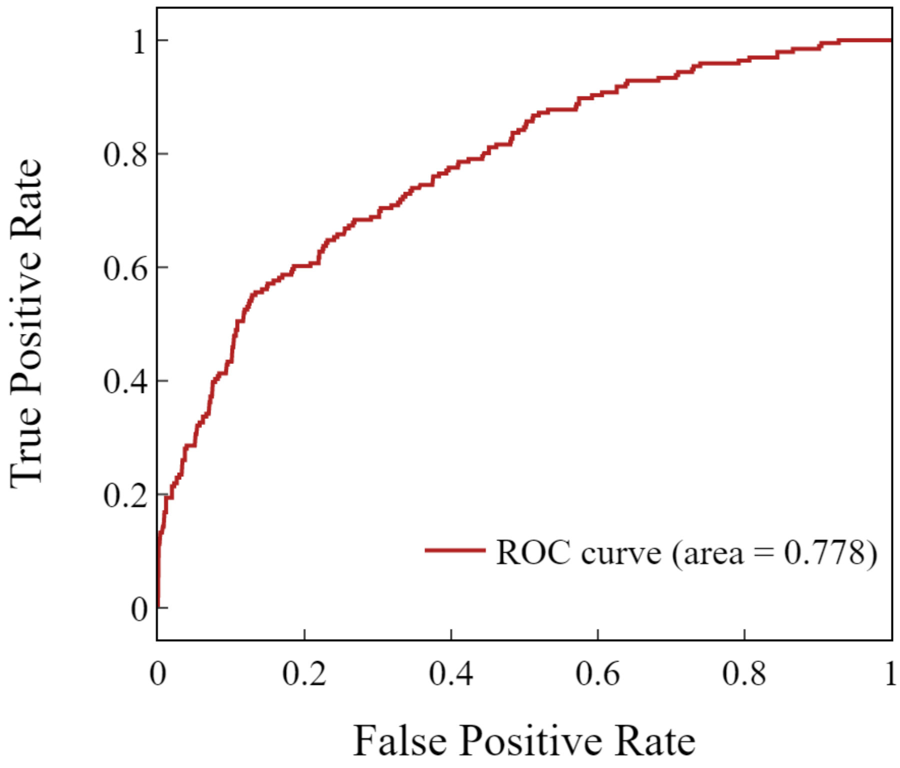

Figure S4. The ROC curve of 1st and 2nd PCA components.

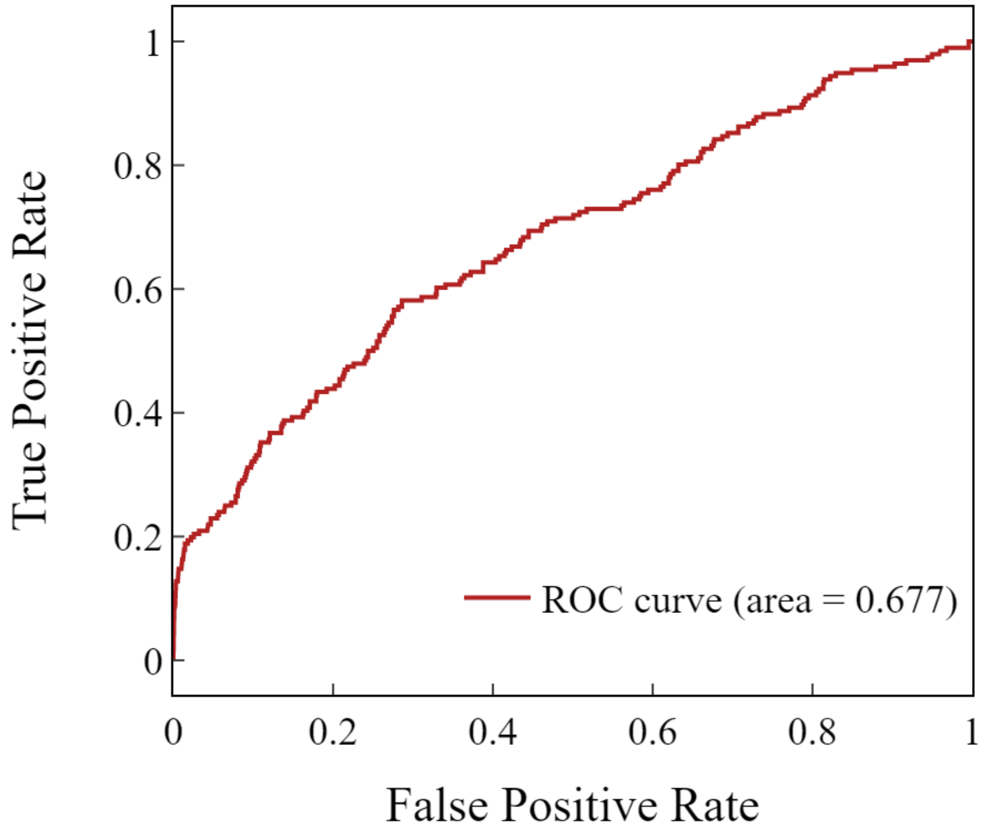

Figure S5. The ROC curve of 1st and 3rd PCA components.

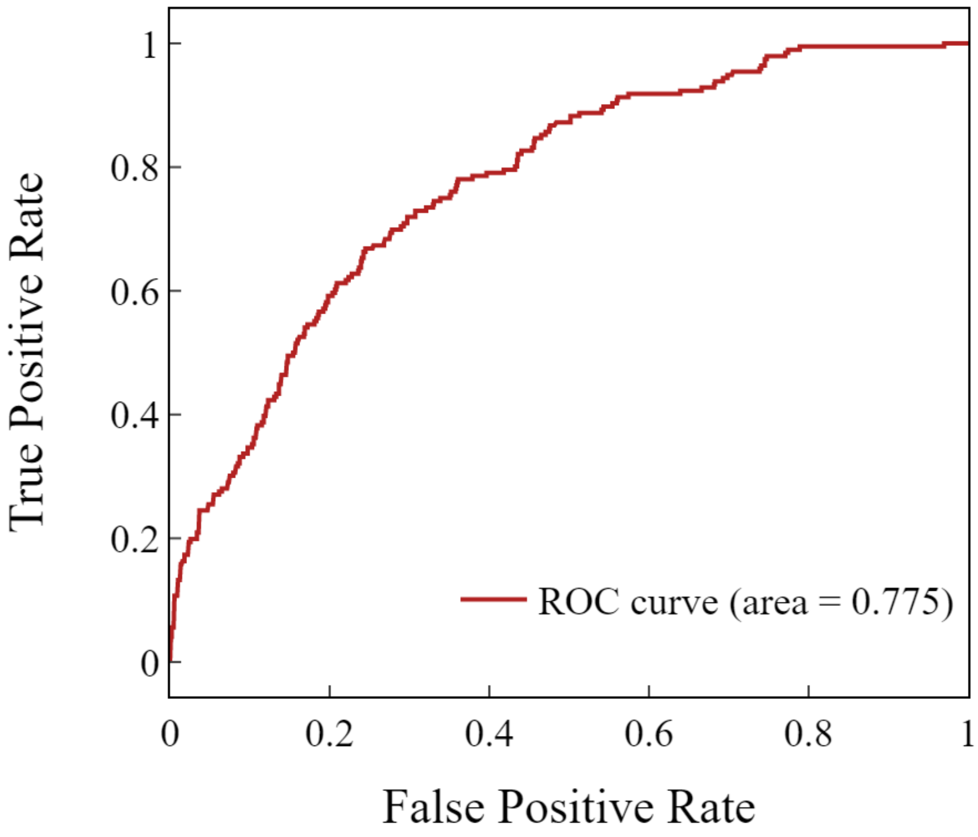

Figure S6. The ROC curve of 1st and 4th PCA components.

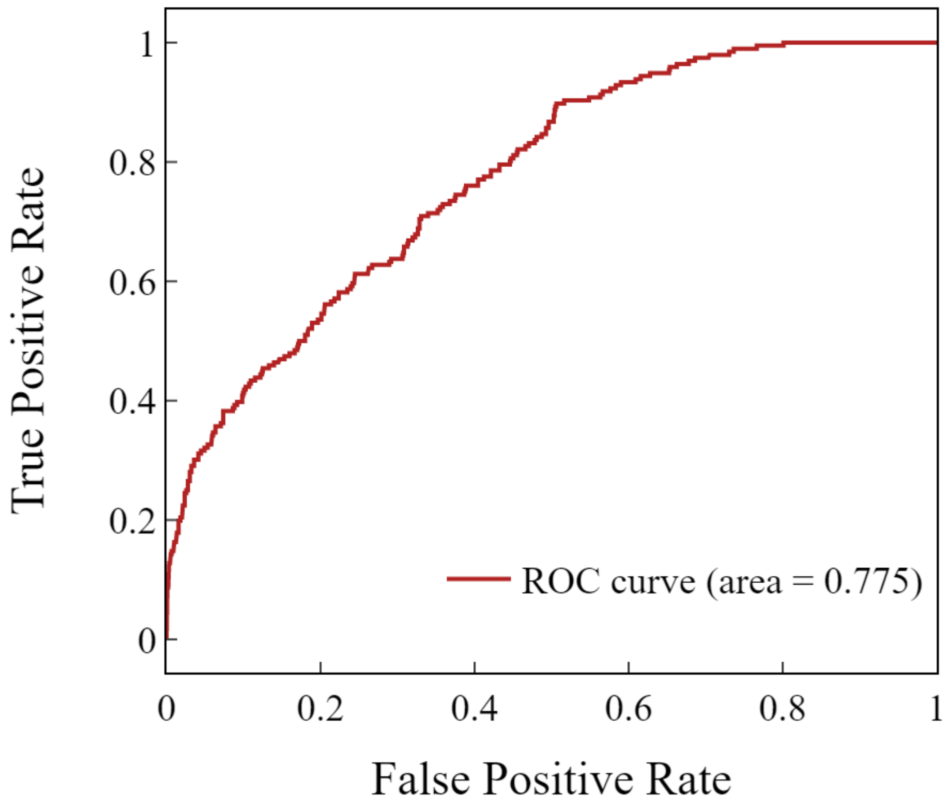

Figure S7. The ROC curve of 1st and 5th PCA components.

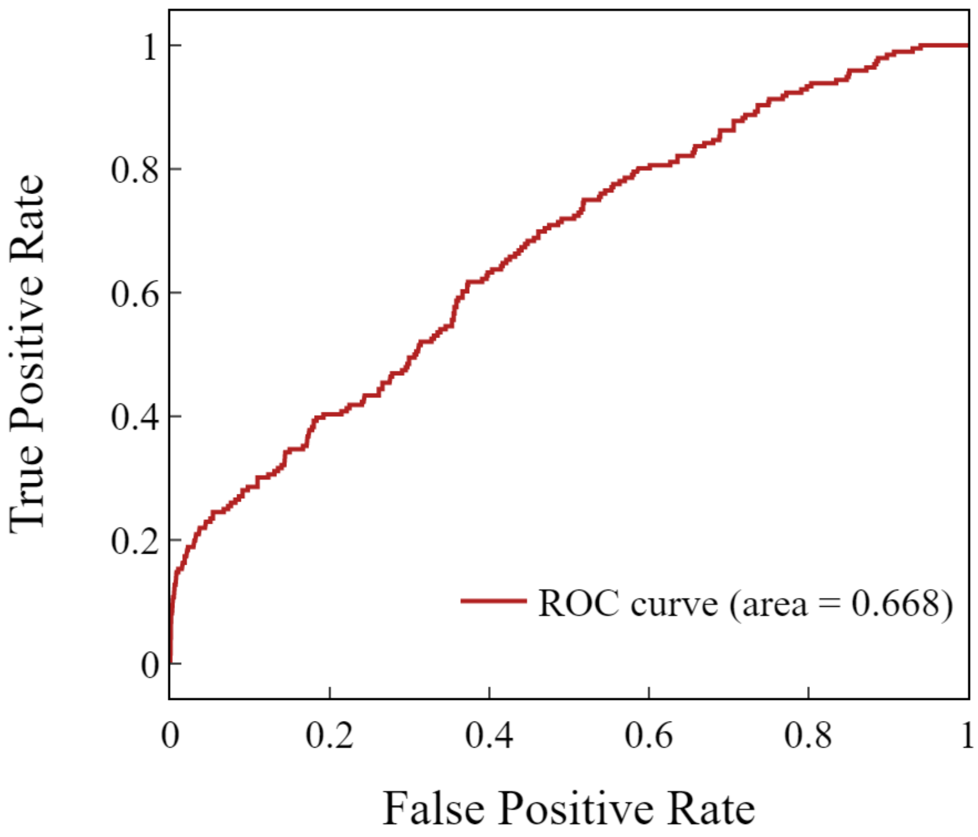

Figure S8. The ROC curve of 1st and 6th PCA components.

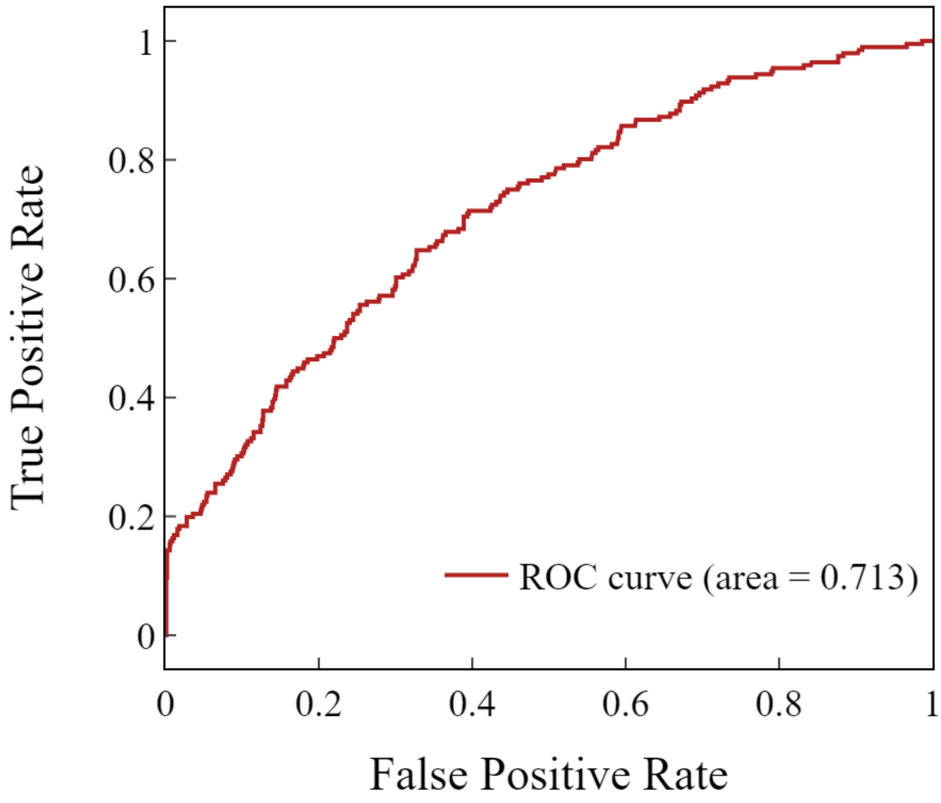

Figure S9. The ROC curve of 1st and 7th PCA components.

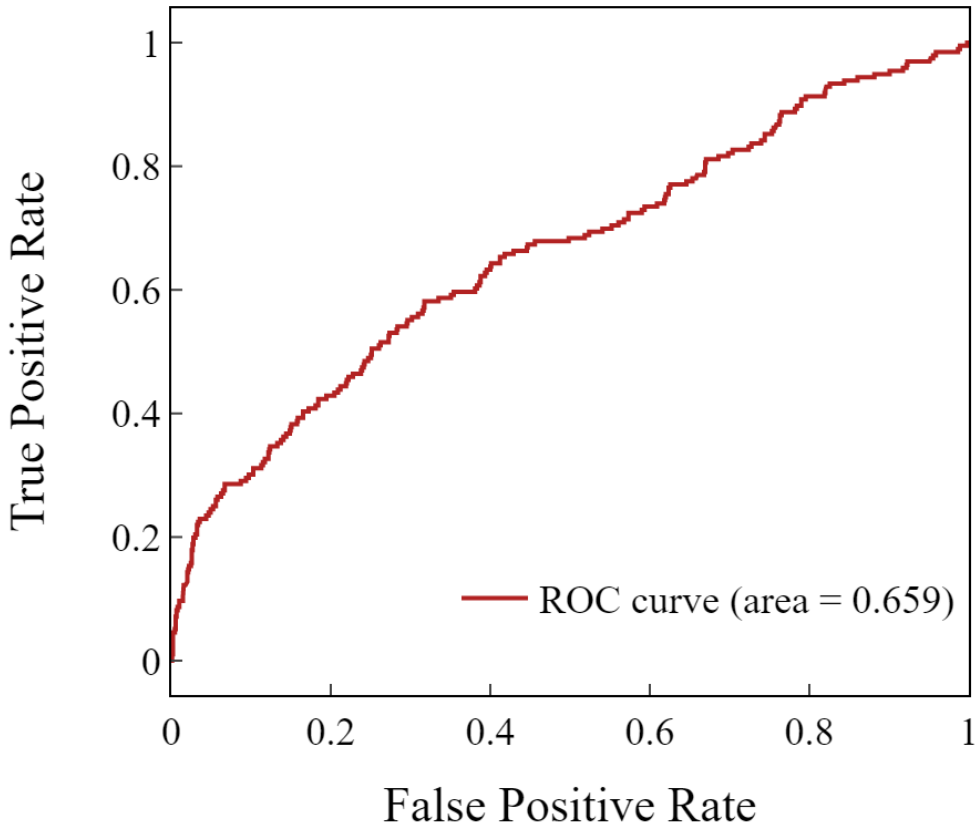

Figure S10. The ROC curve of 1st and 8th PCA components.

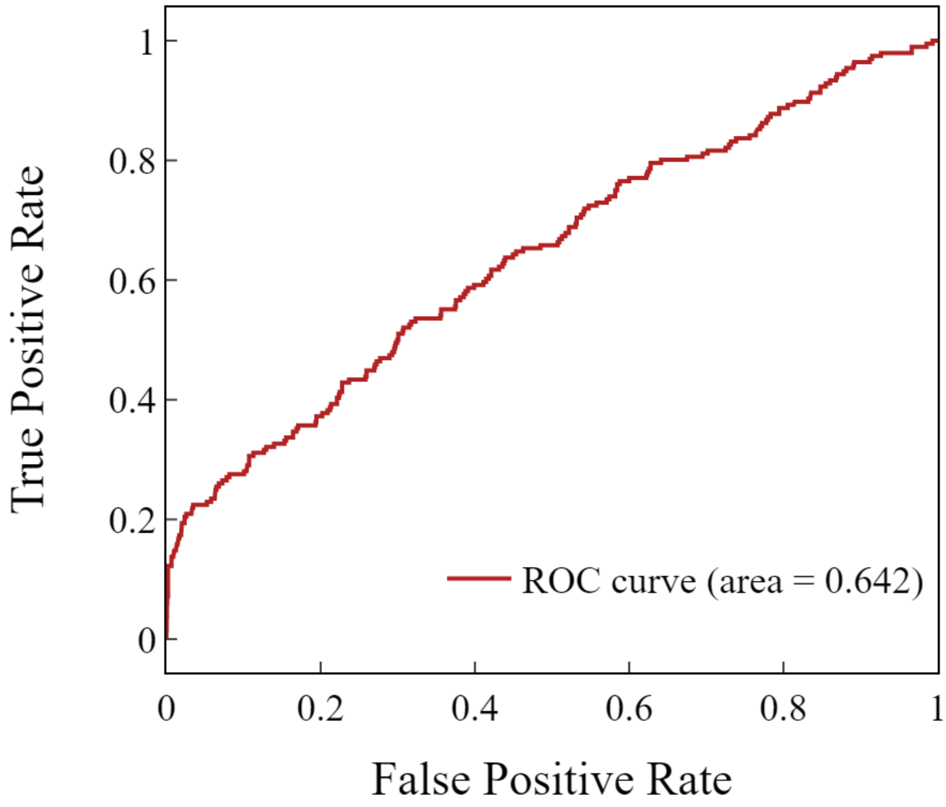

Figure S11. The ROC curve of 1st and 9th PCA components.

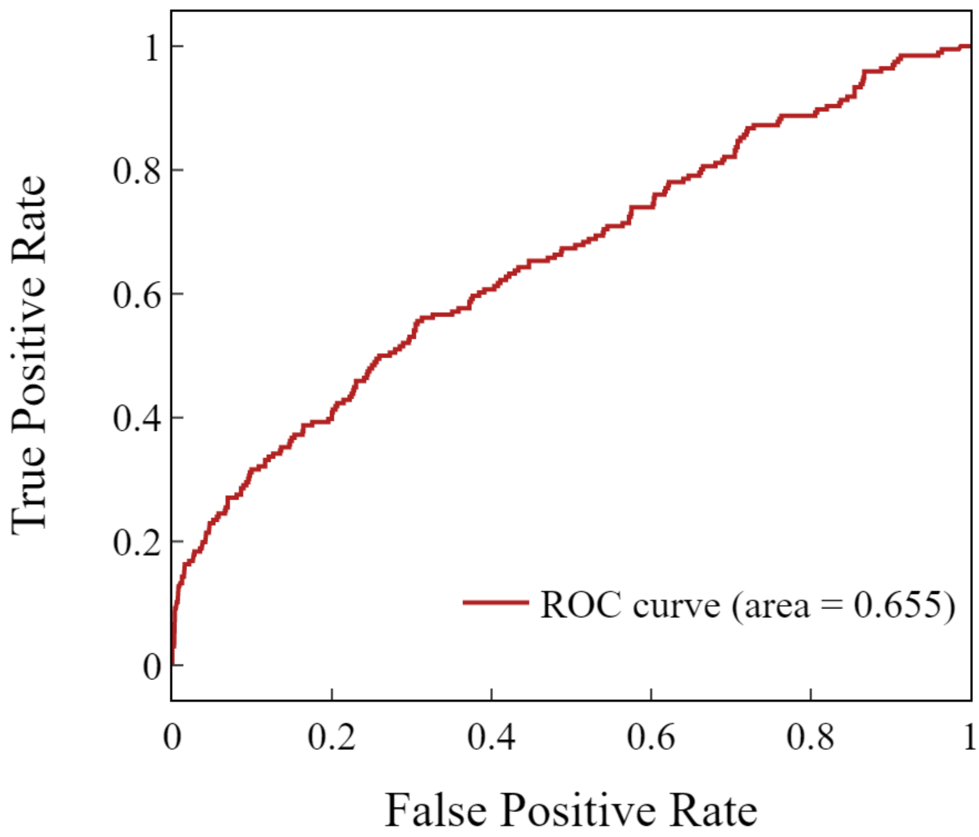

Figure S12. The ROC curve of 1st and 10th PCA components.

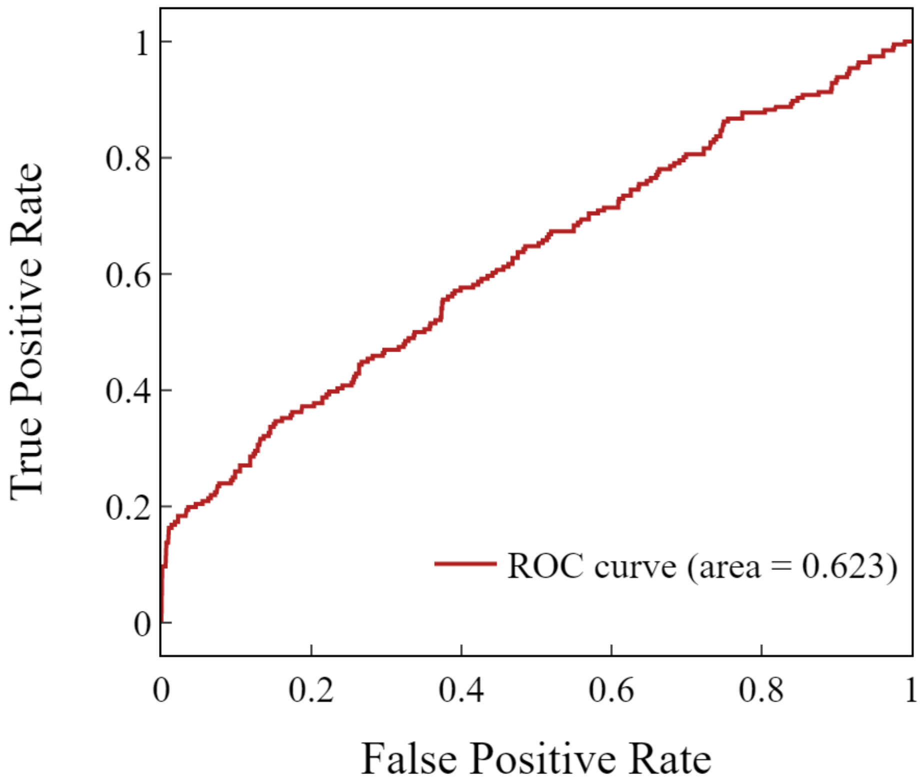

Figure S13. The ROC curve of 1th and 11th PCA components.

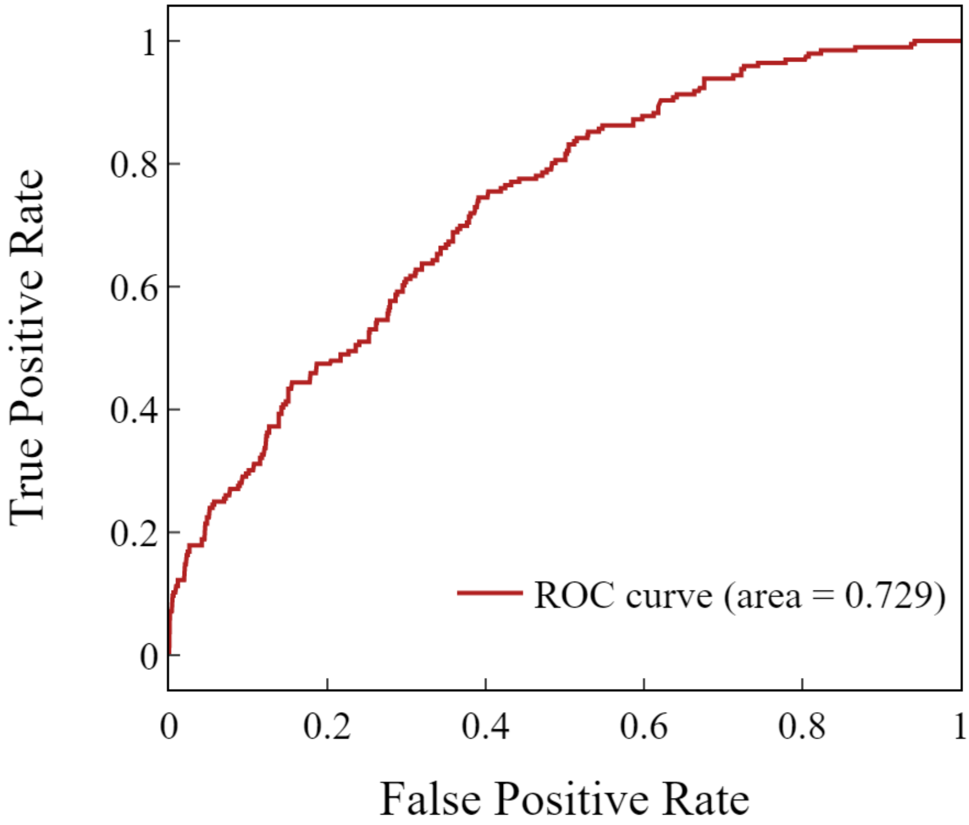

Figure S14. The ROC curve of 2nd and 3rd PCA components.

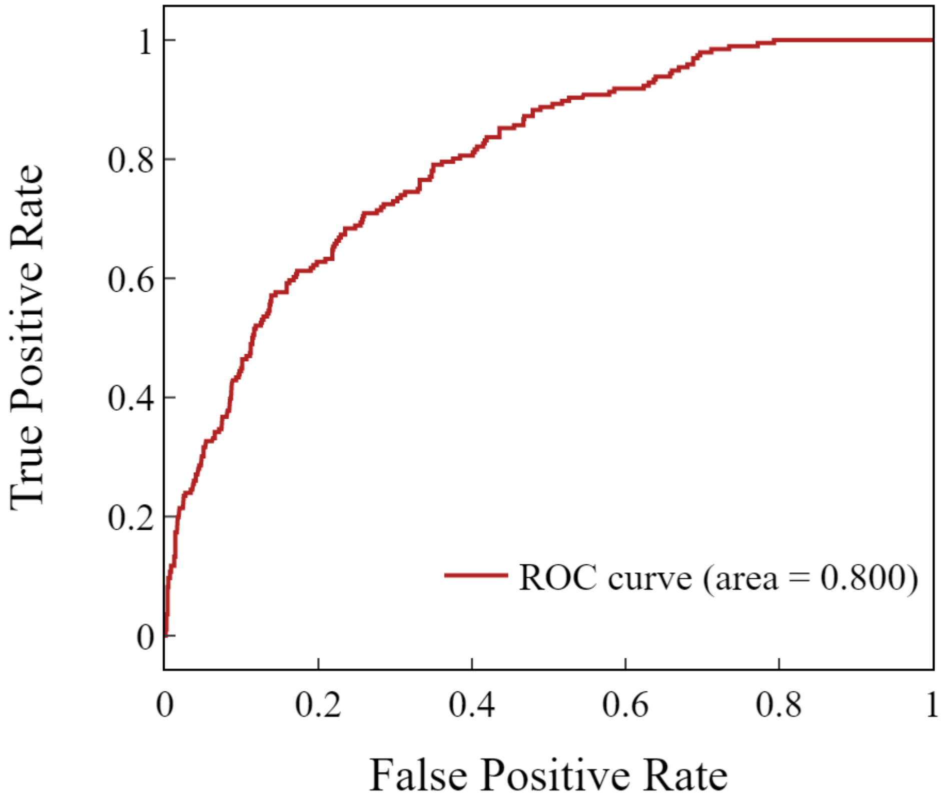

Figure S15. The ROC curve of 2nd and 4th PCA components.

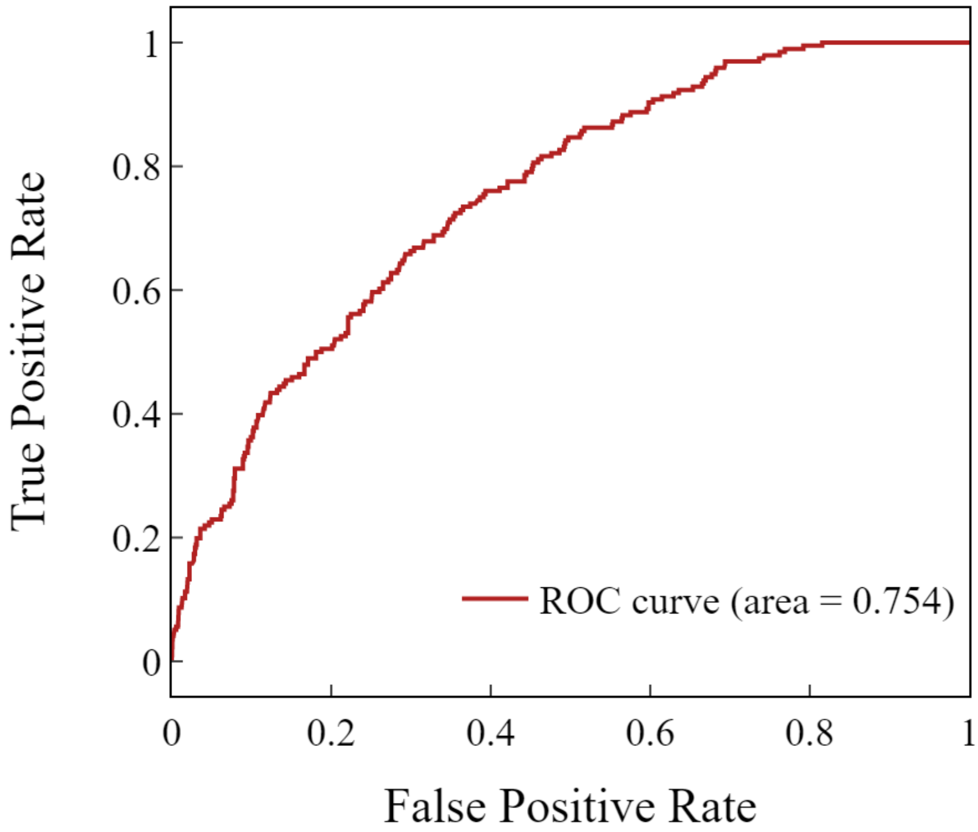

Figure S16. The ROC curve of 2nd and 5th PCA components.

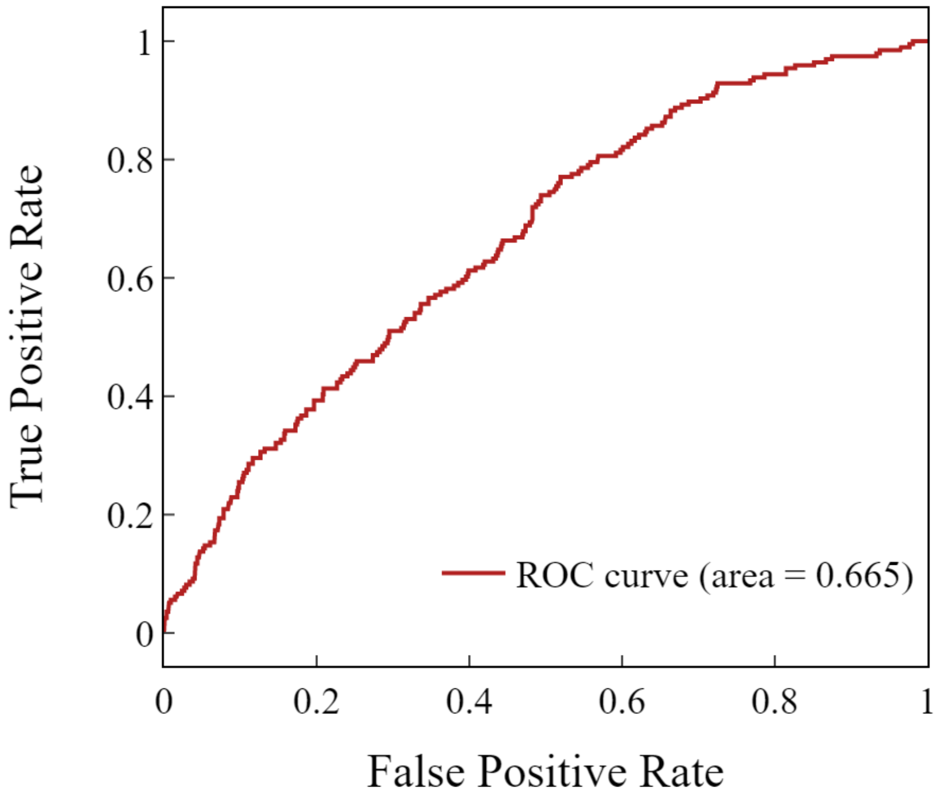

Figure S17. The ROC curve of 2nd and 6th PCA components.

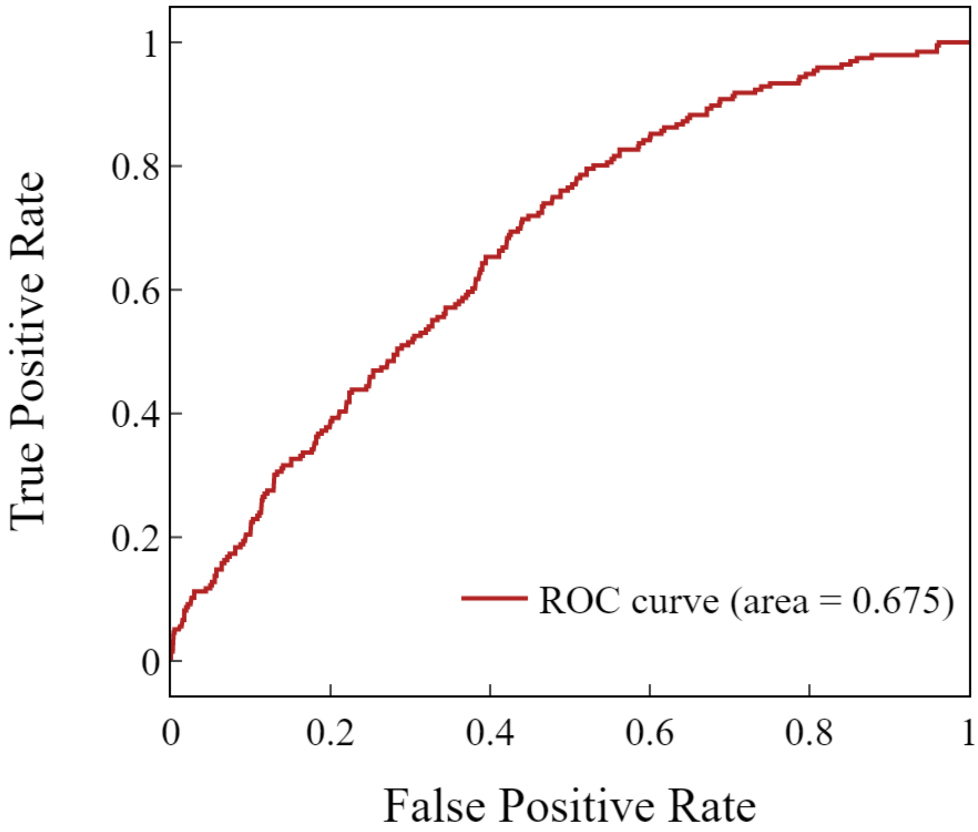

Figure S18. The ROC curve of 2nd and 7th PCA components.

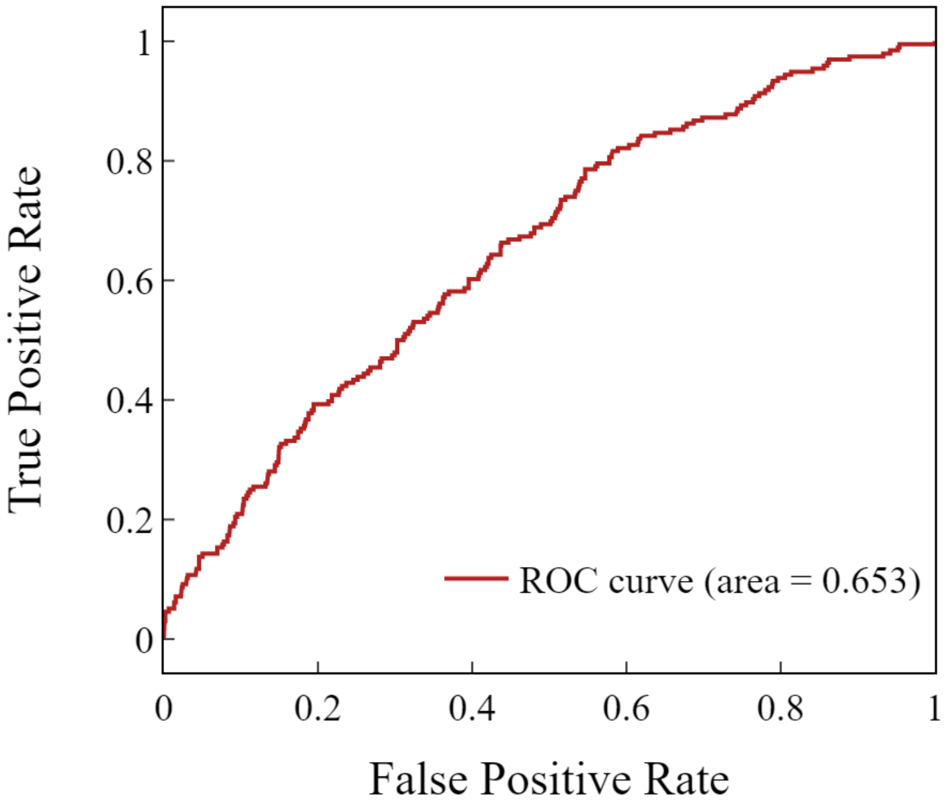

Figure S19. The ROC curve of 2nd and 8th PCA components.

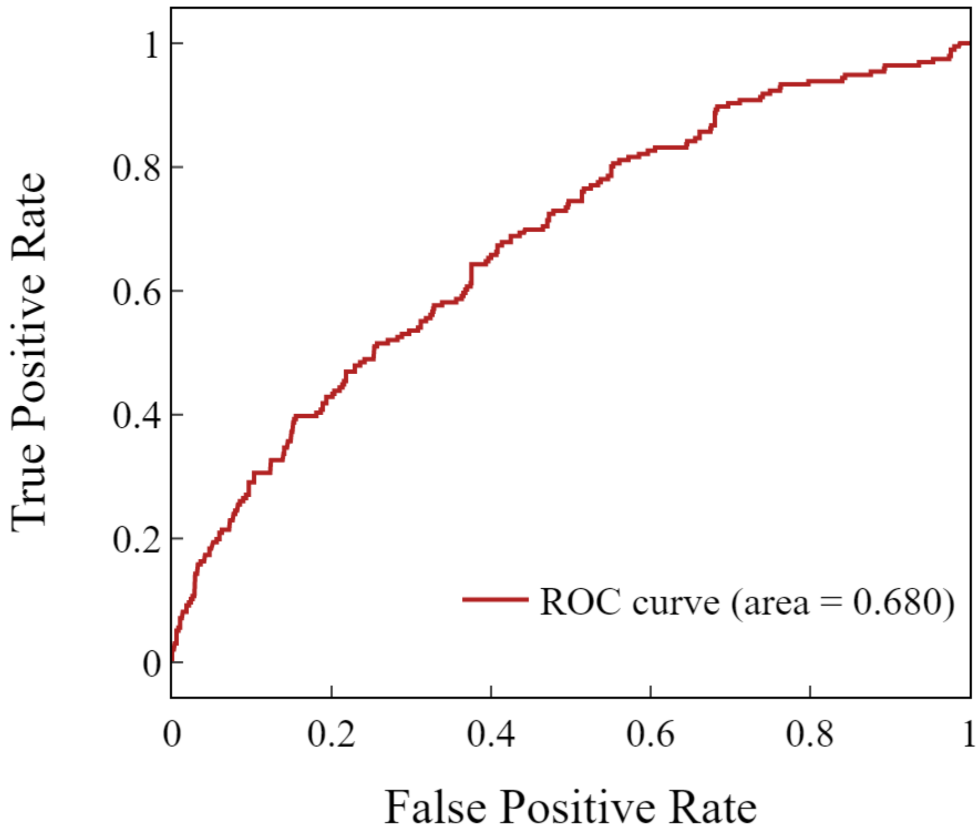

Figure S20. The ROC curve of 2nd and 9th PCA components.

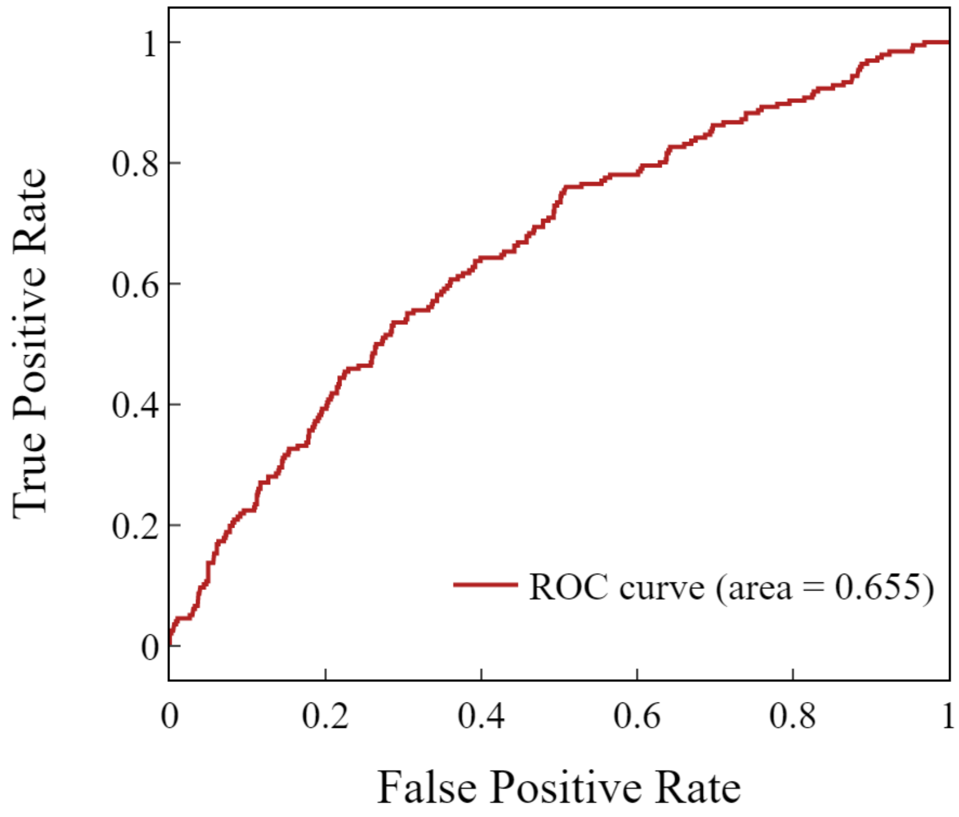

Figure S21. The ROC curve of 2nd and 10th PCA components.

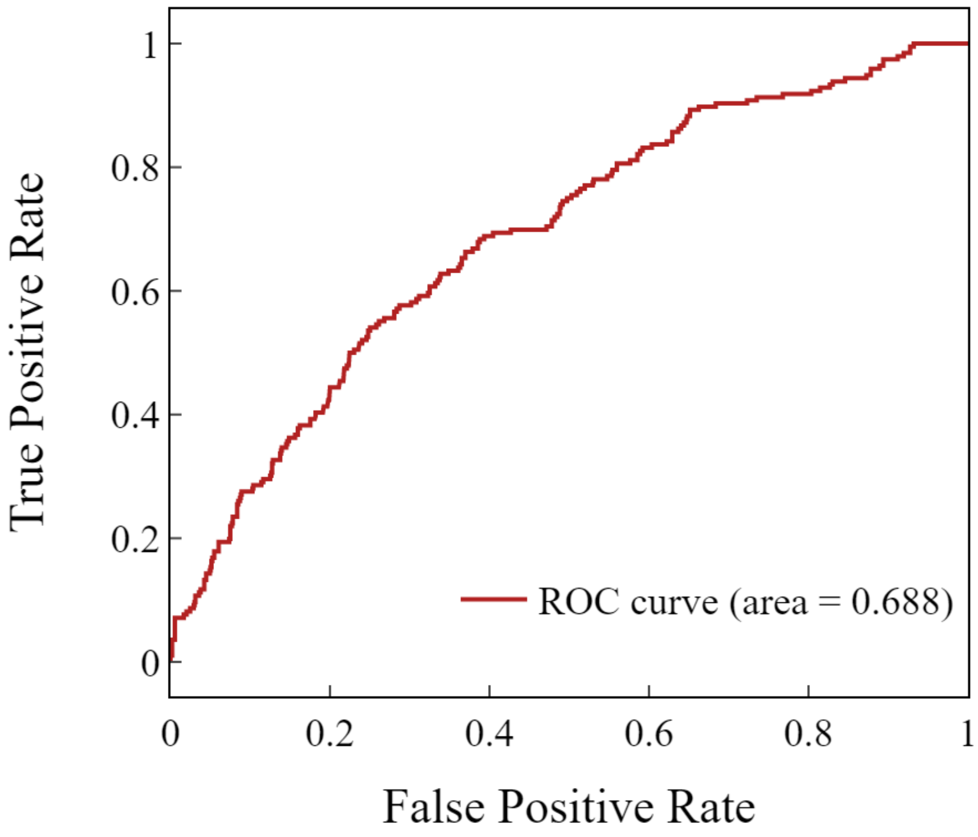

Figure S22. The ROC curve of 2th and 11th PCA components.

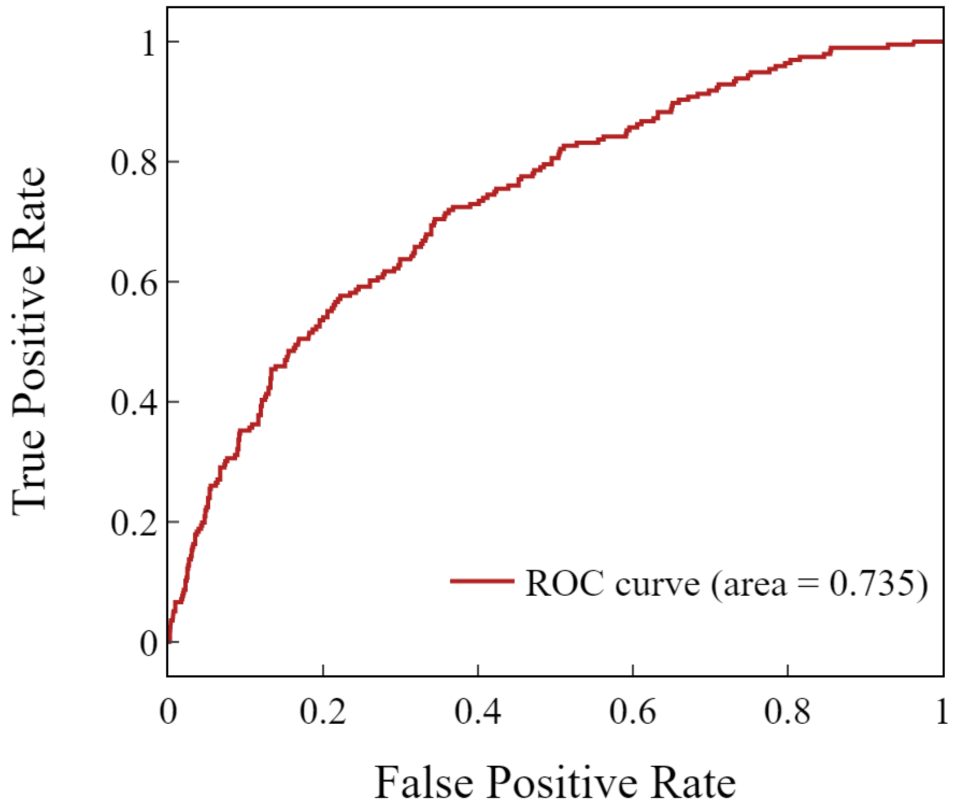

Figure S23. The ROC curve of 3rd and 4th PCA components.

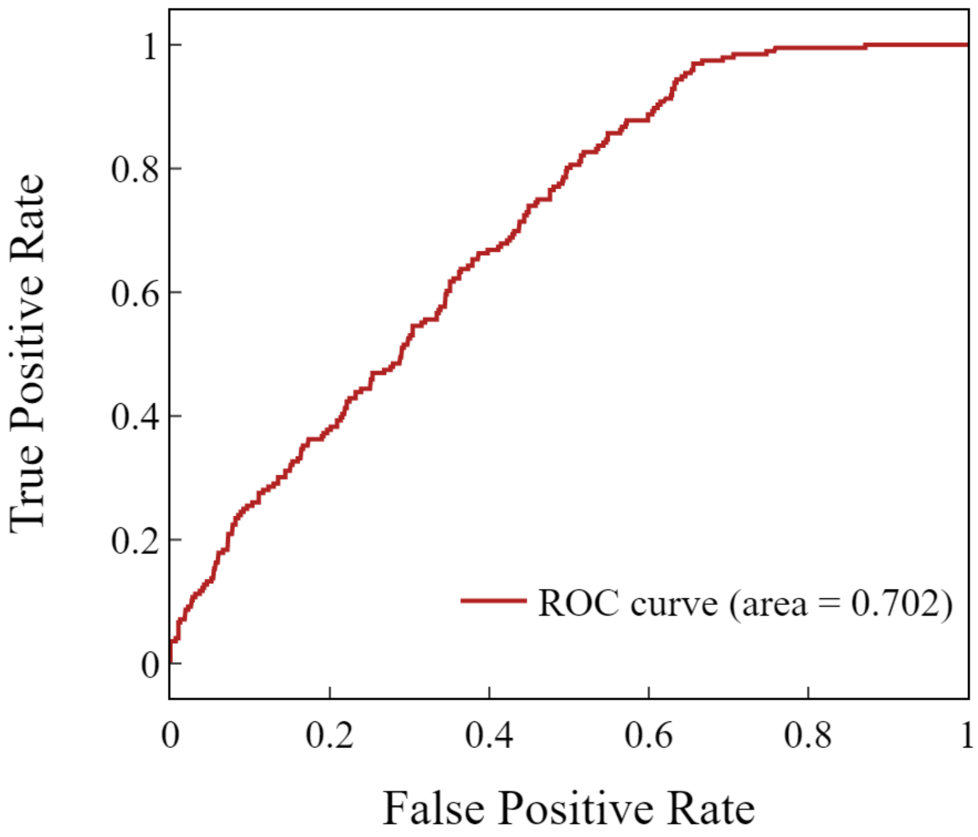

Figure S24. The ROC curve of 3rd and 5th PCA components.

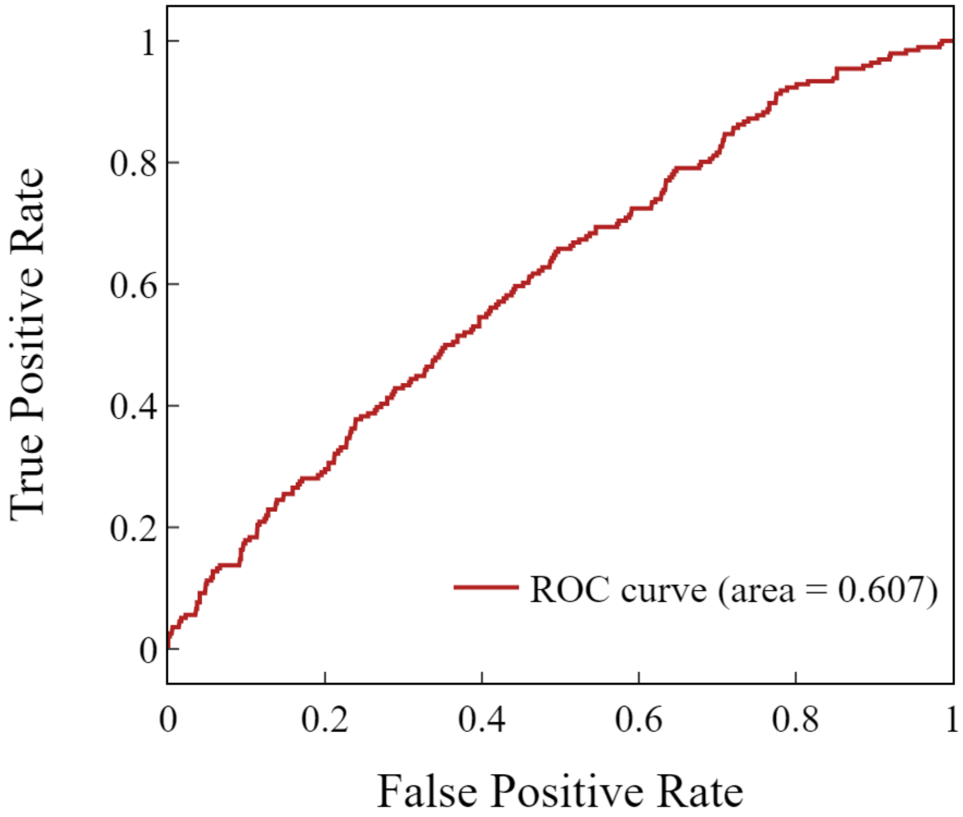

Figure S25. The ROC curve of 3rd and 6th PCA components.

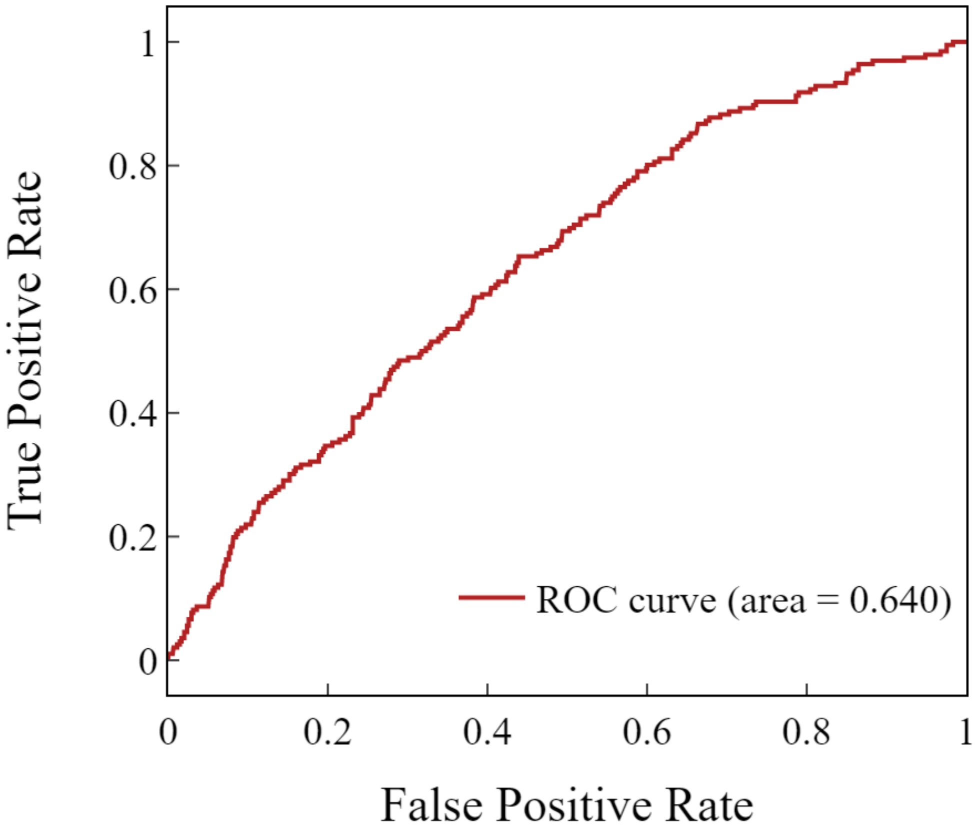

Figure S26. The ROC curve of 3rd and 7th PCA components.

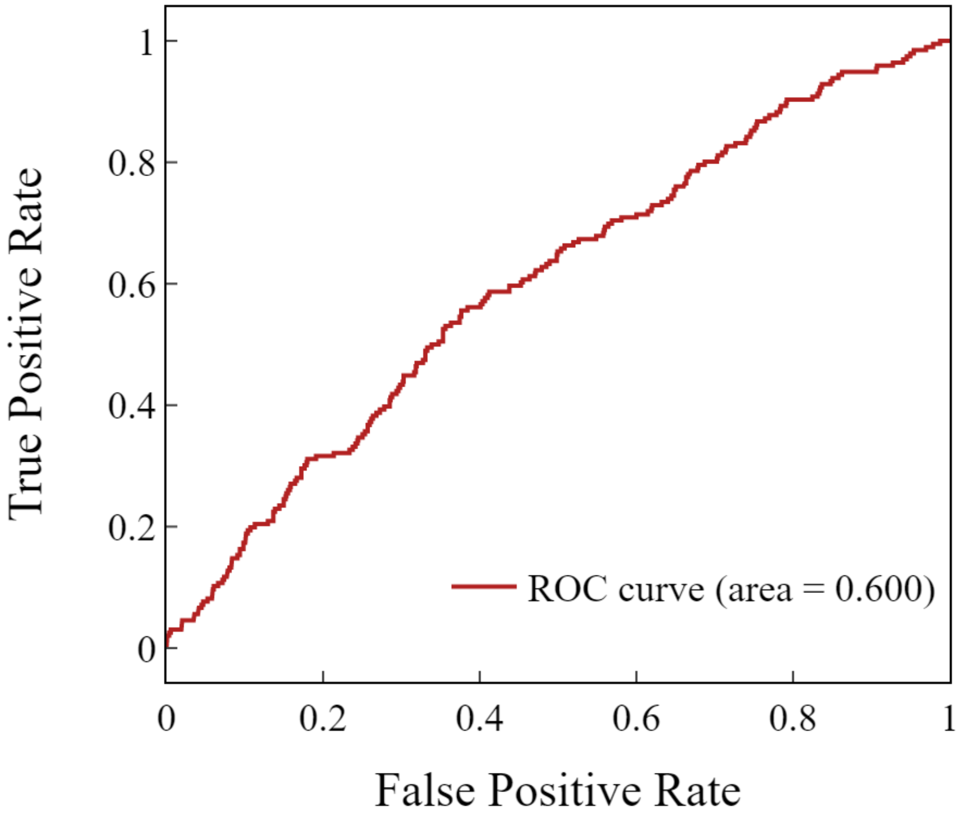

Figure S27. The ROC curve of 3rd and 8th PCA components.

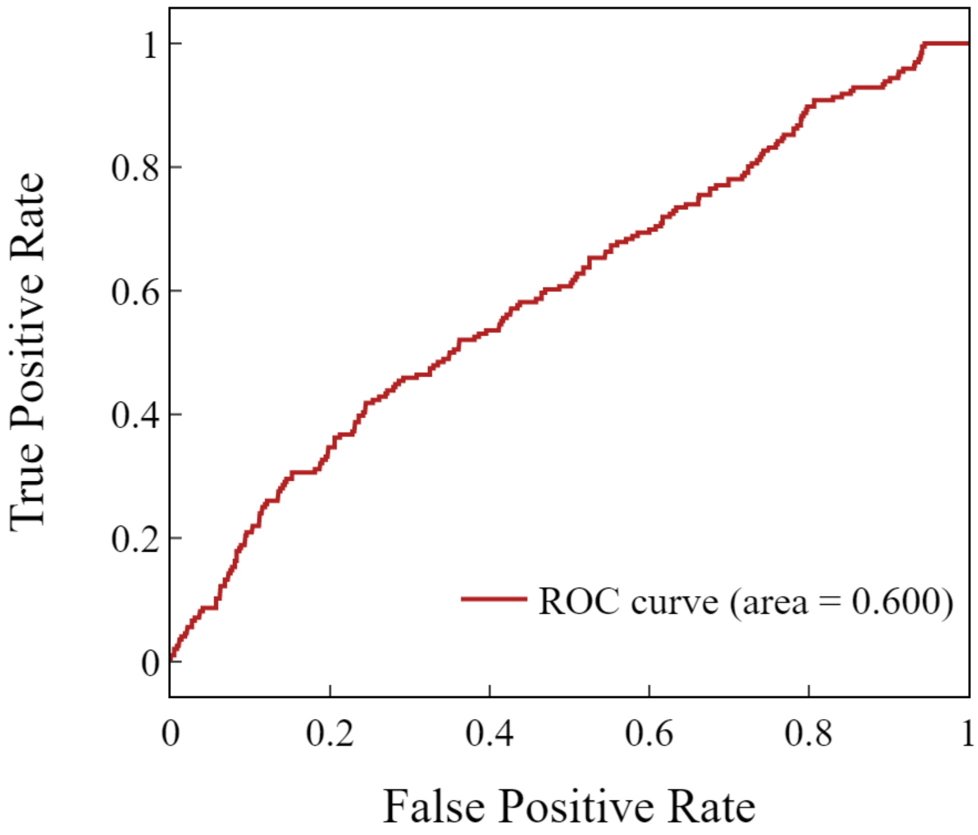

Figure S28. The ROC curve of 3rd and 9th PCA components.

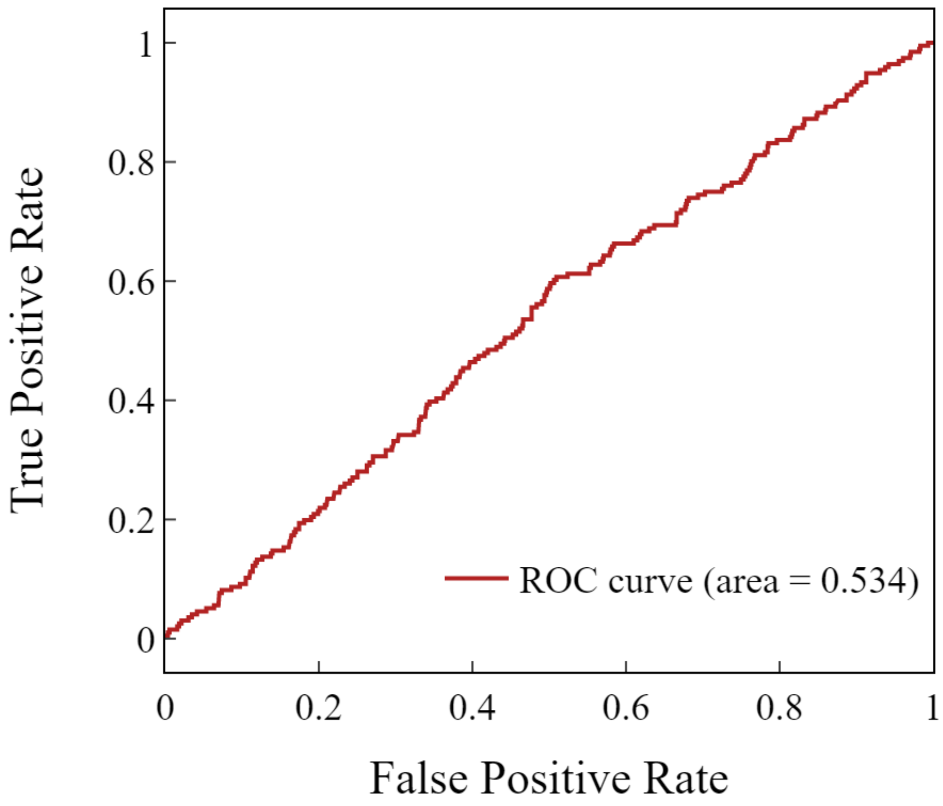

Figure S29. The ROC curve of 3rd and 10th PCA components.

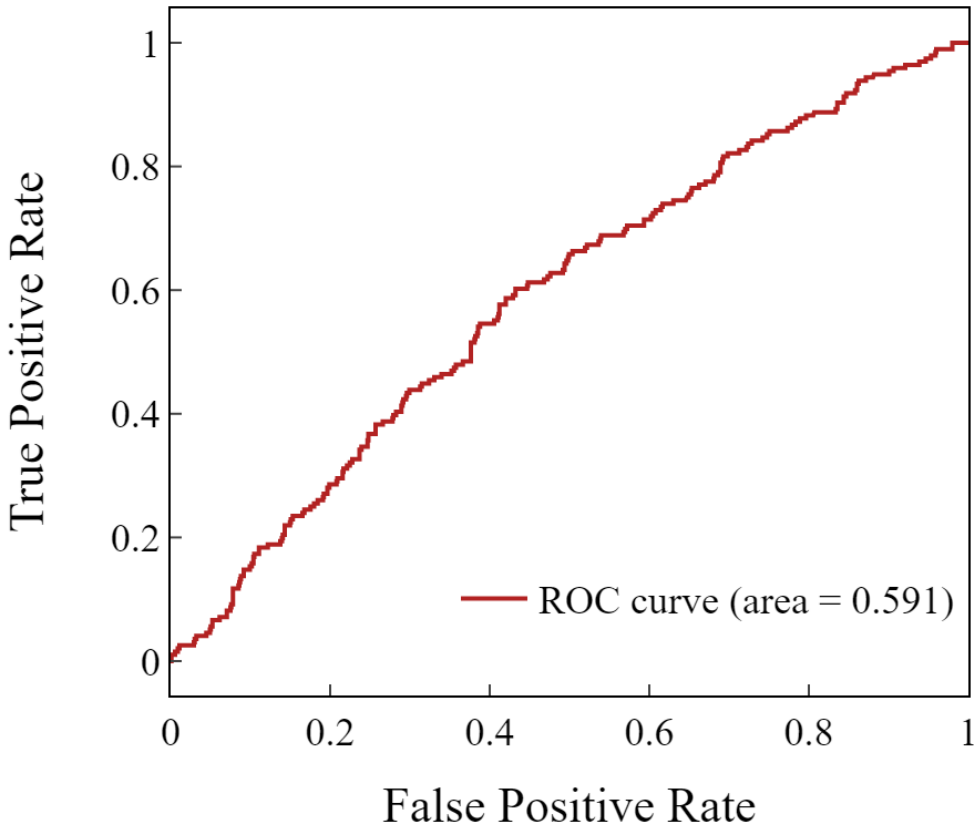

Figure S30. The ROC curve of 3rd and 11th PCA components.

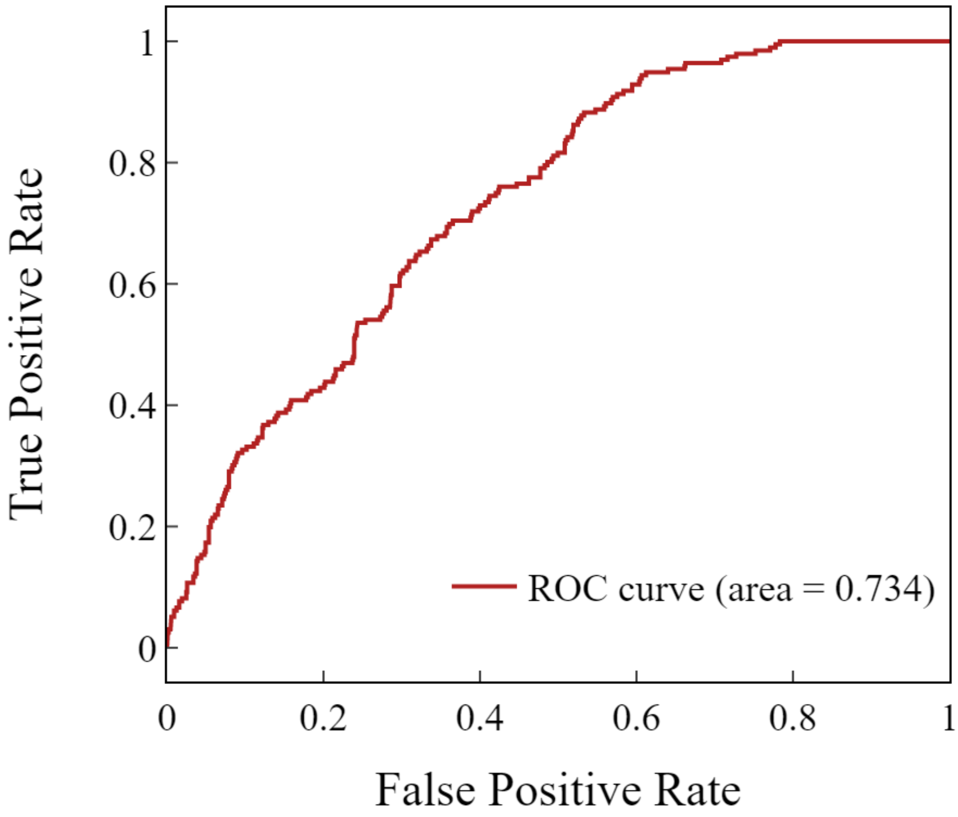

Figure S31. The ROC curve of 4th and 5th PCA components.

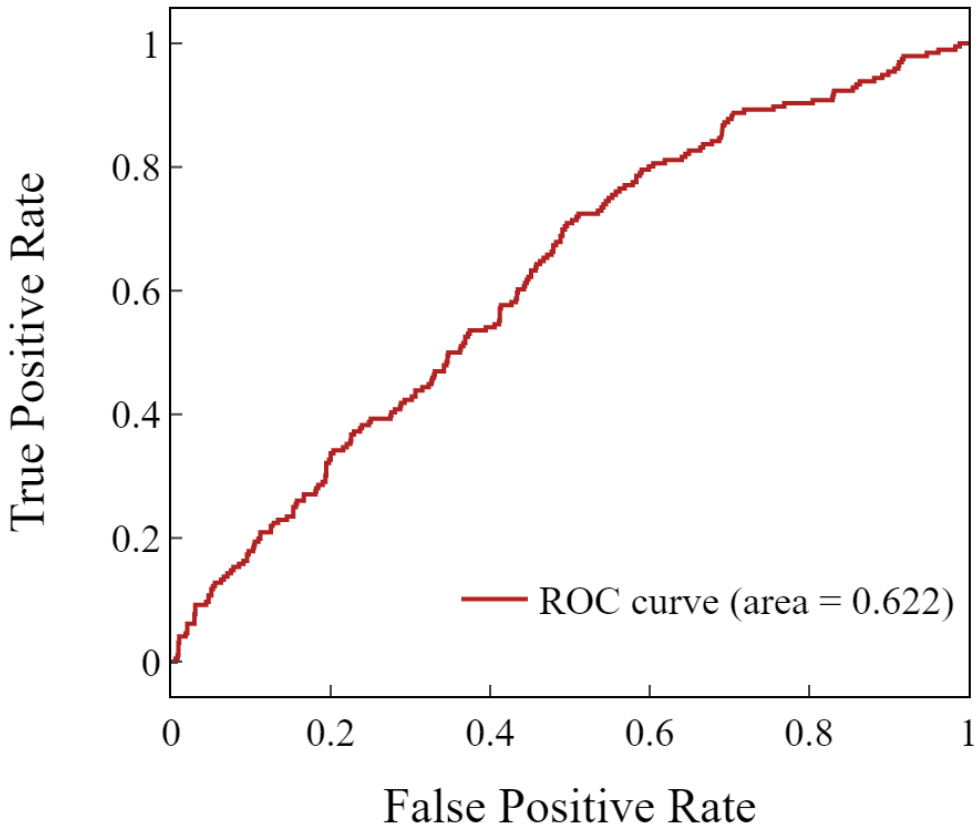

Figure S32. The ROC curve of 4th and 6th PCA components.

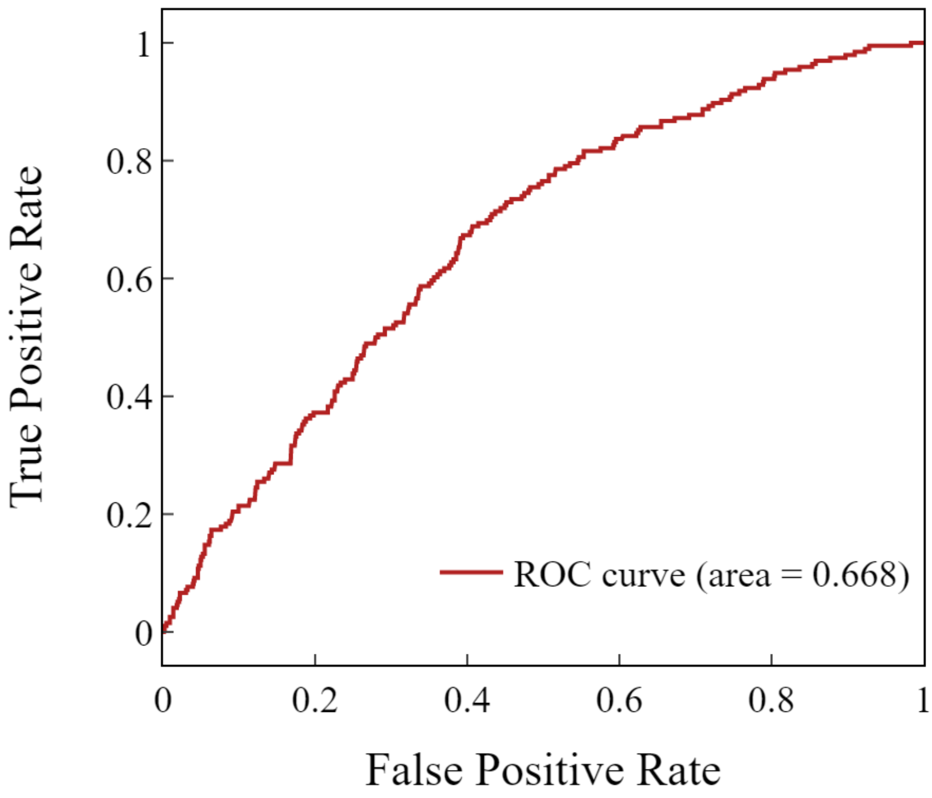

Figure S33. The ROC curve of 4th and 7th PCA components.

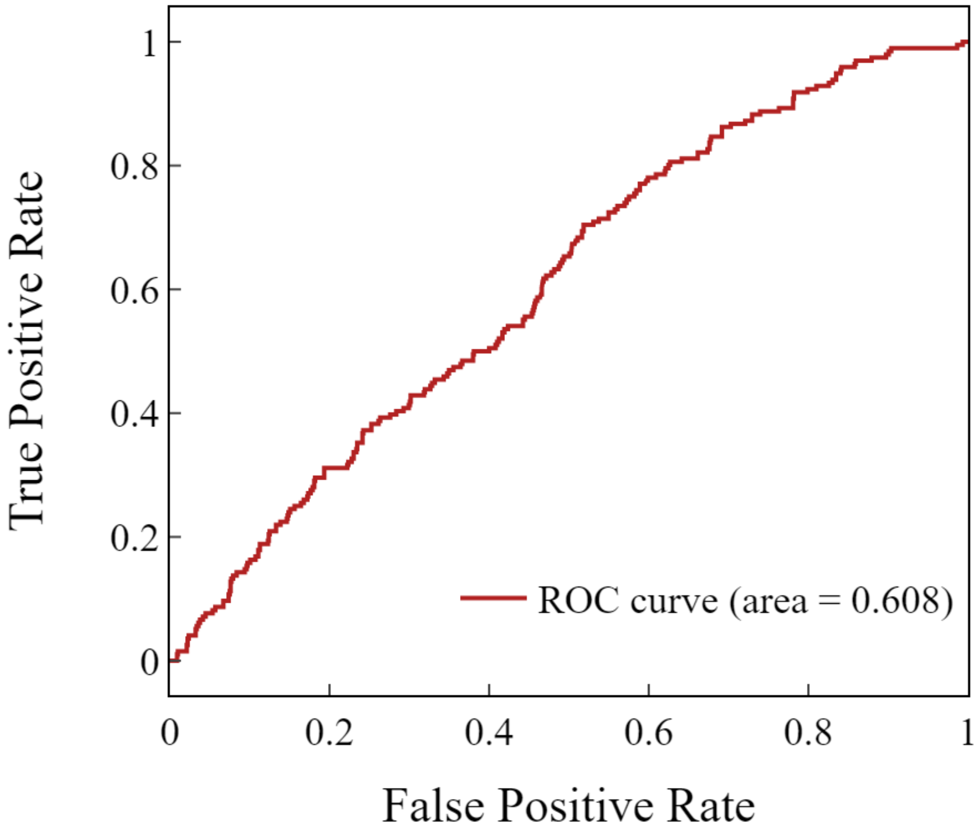

Figure S34. The ROC curve of 4th and 8th PCA components.

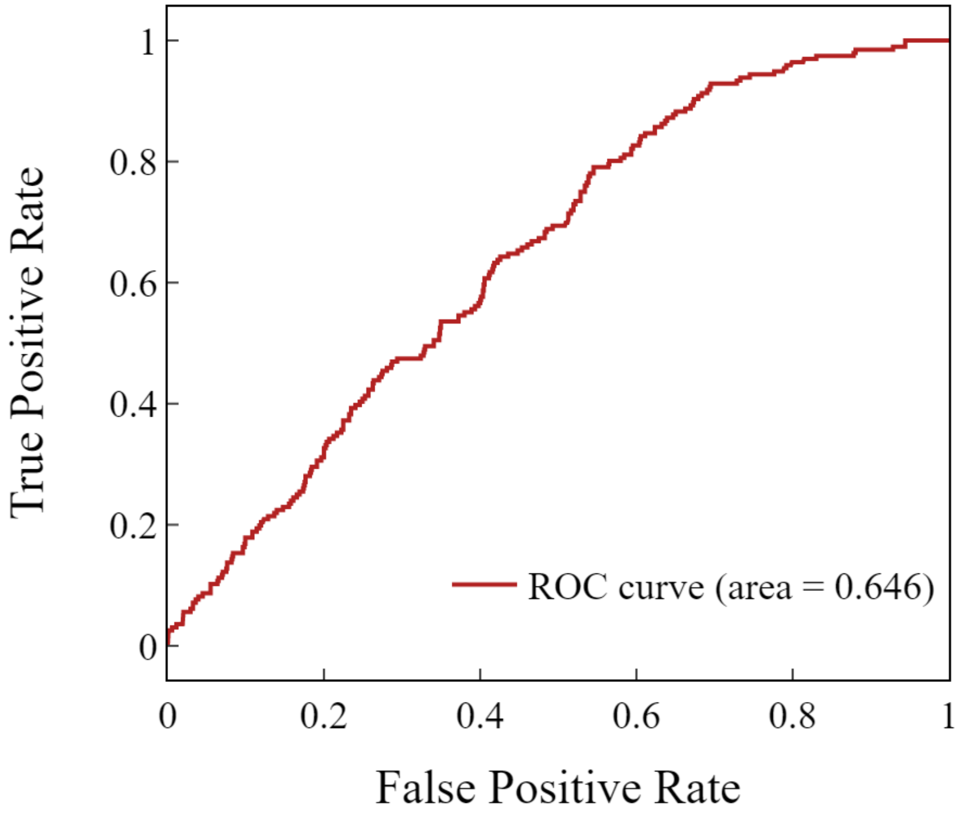

Figure S35. The ROC curve of 4th and 9th PCA components.

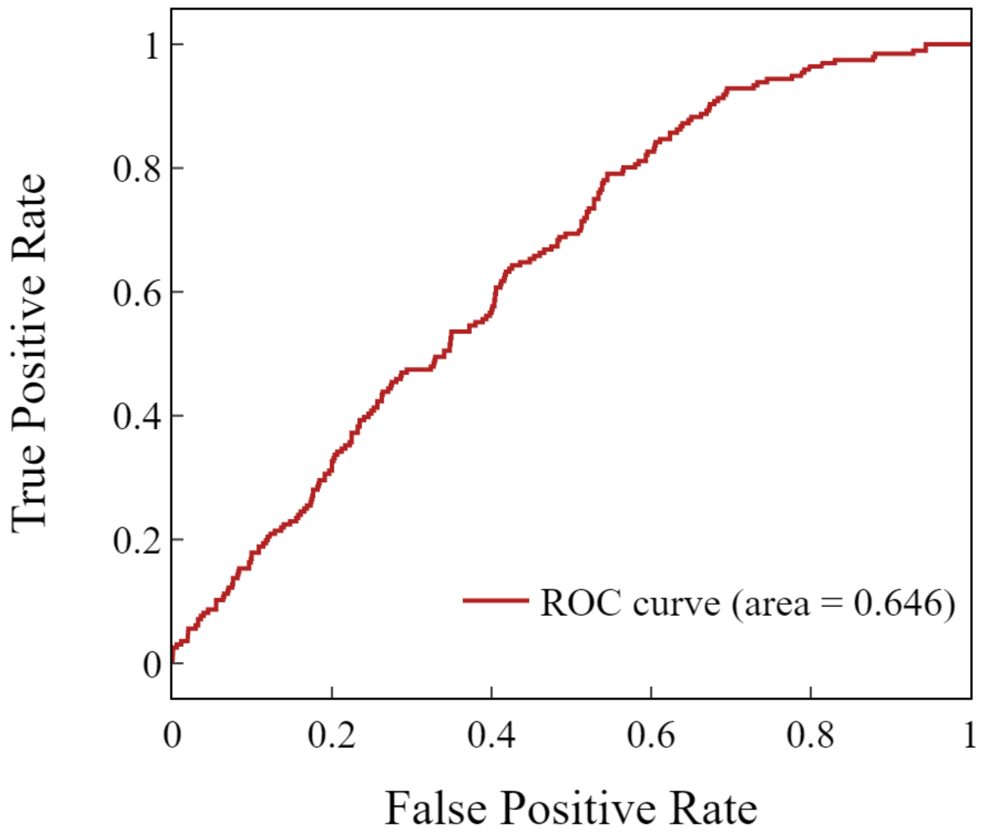

Figure S36. The ROC curve of 4th and 10th PCA components.

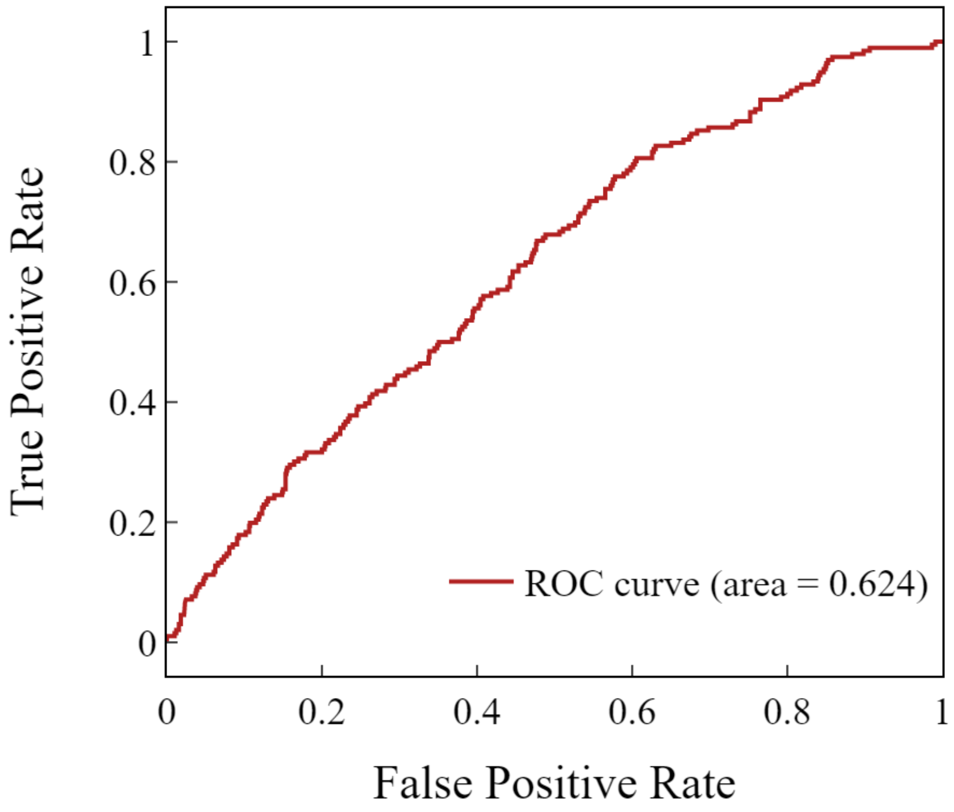

Figure S37. The ROC curve of 4th and 11th PCA components.

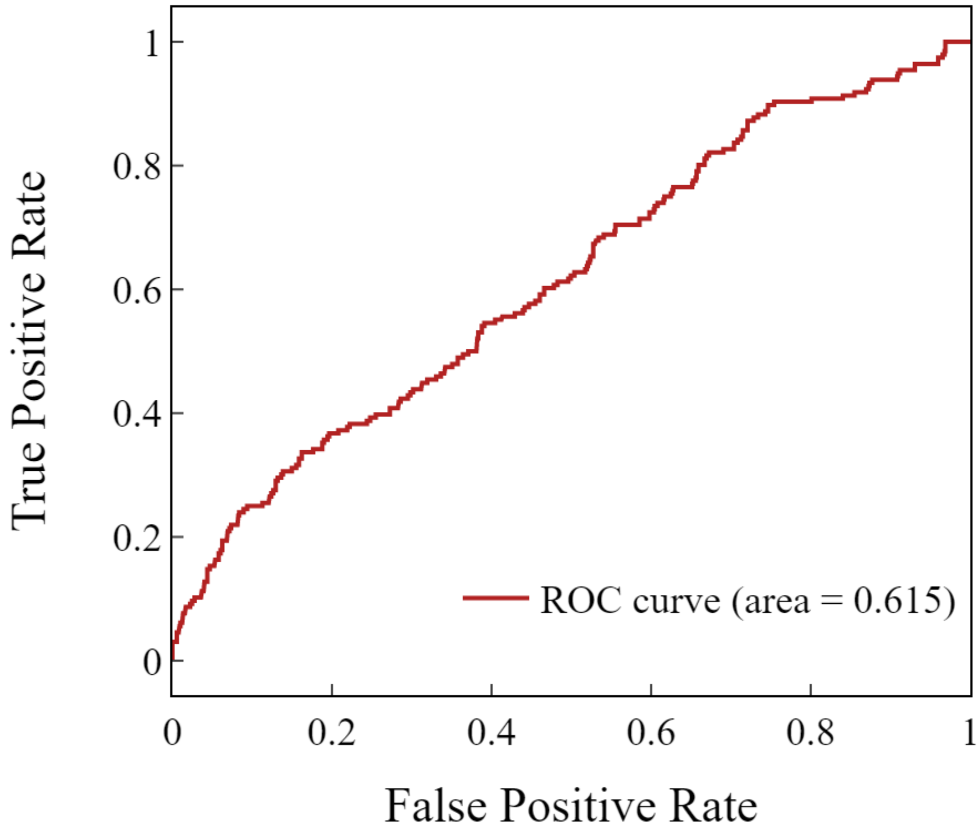

Figure S38. The ROC curve of 5th and 6th PCA components.

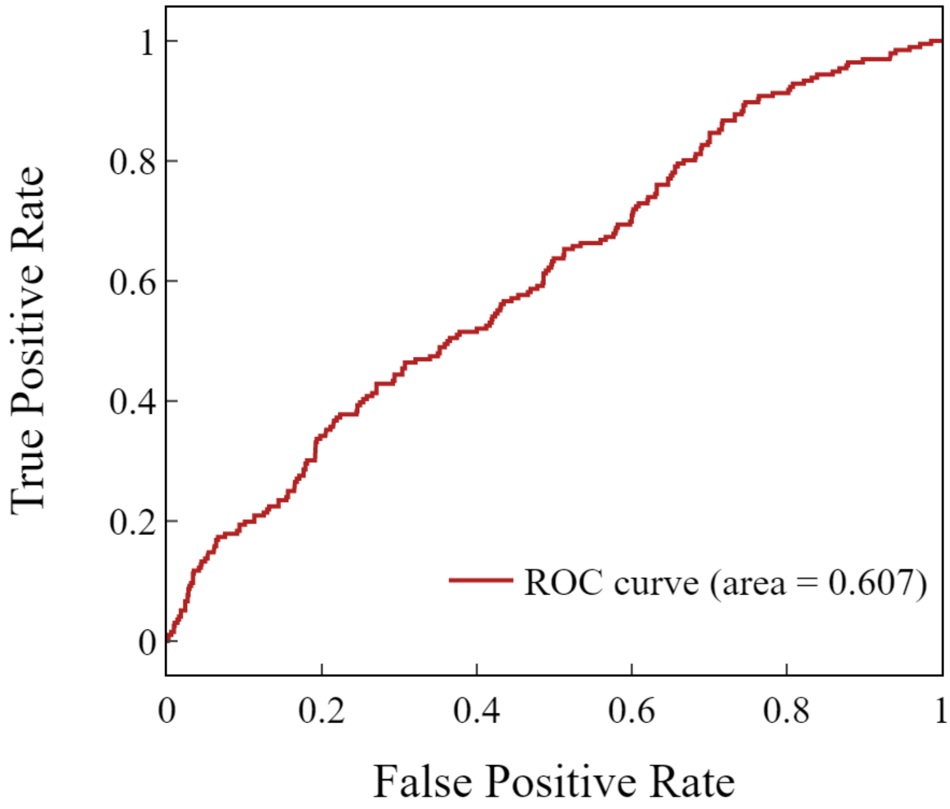

Figure S39. The ROC curve of 5th and 7th PCA components.

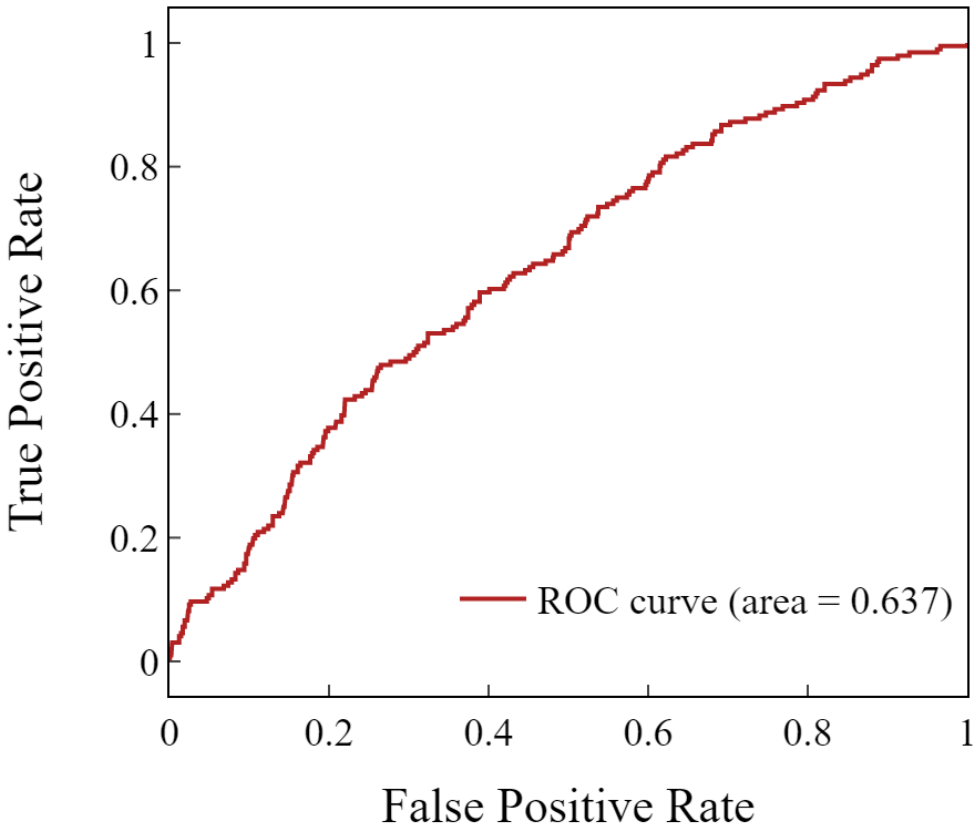

Figure S40. The ROC curve of 5th and 8th PCA components.

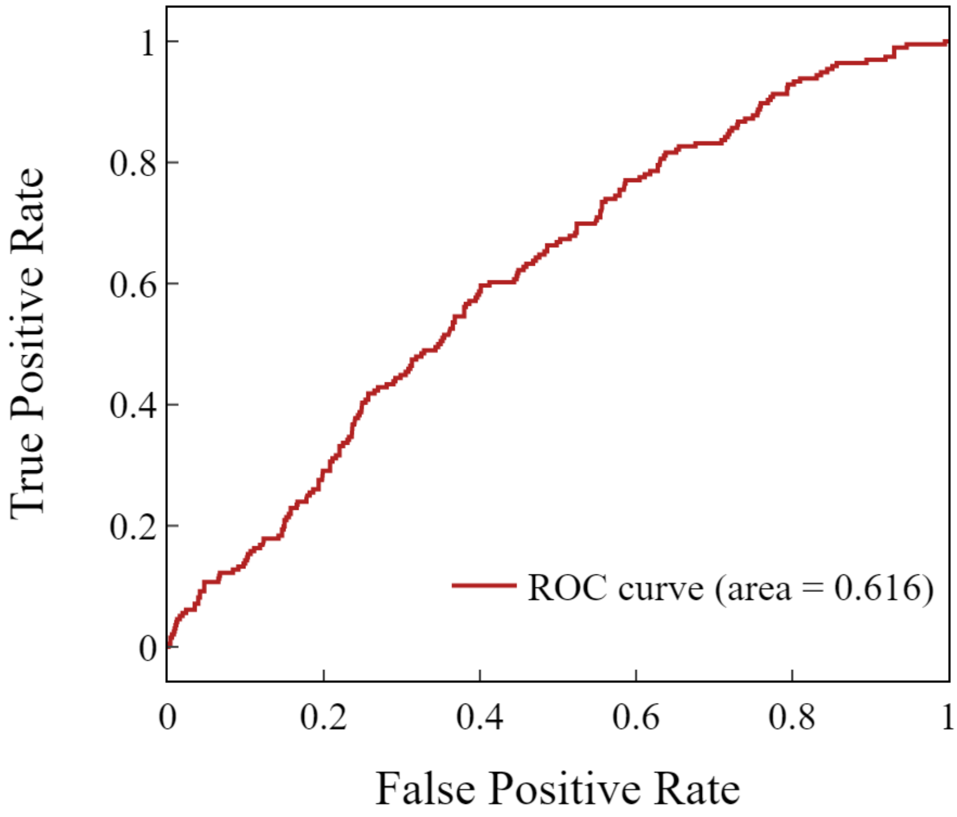

Figure S41. The ROC curve of 5th and 9th PCA components.

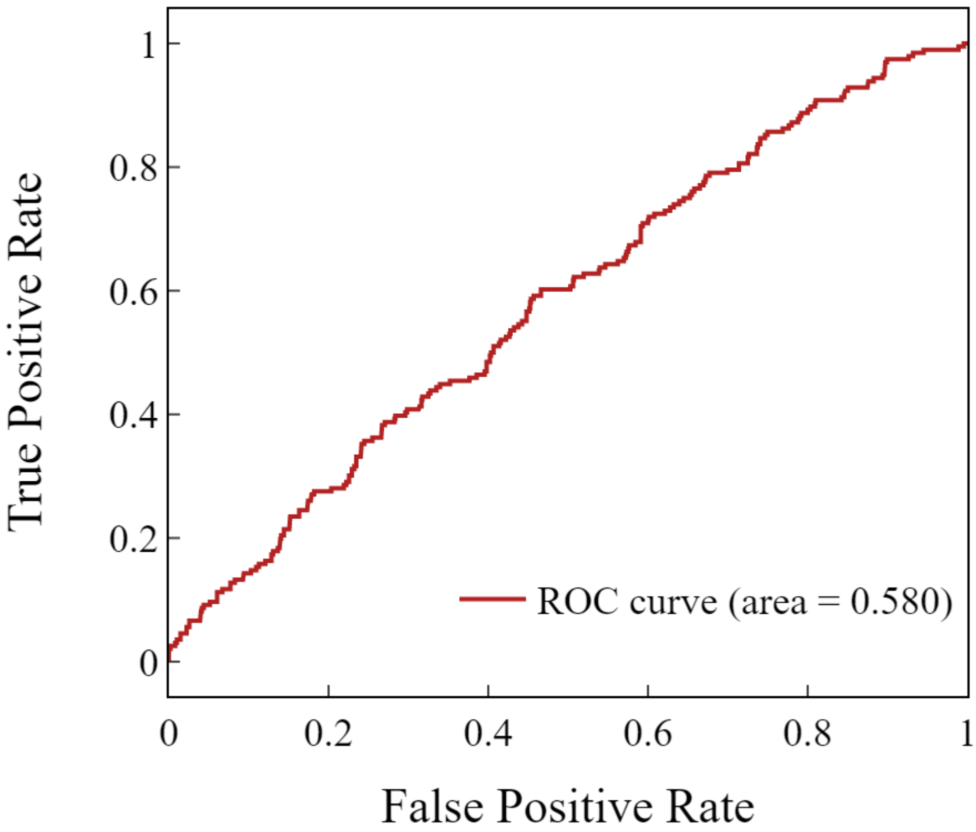

Figure S42. The ROC curve of 5th and 10th PCA components.

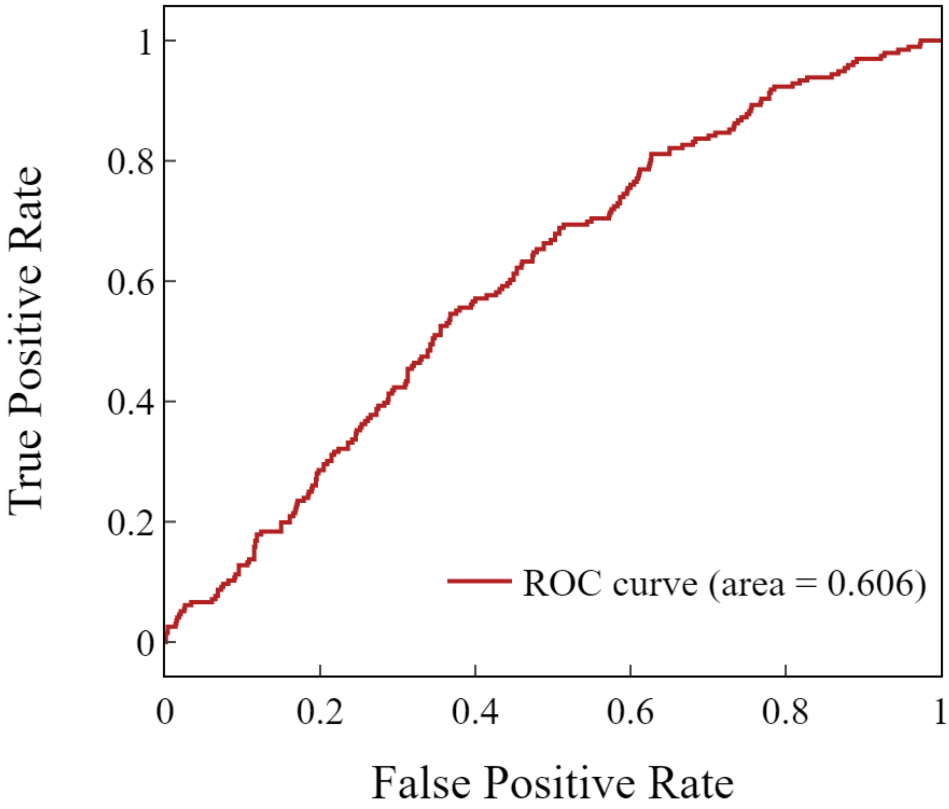

Figure S43. The ROC curve of 5th and 11th PCA components.

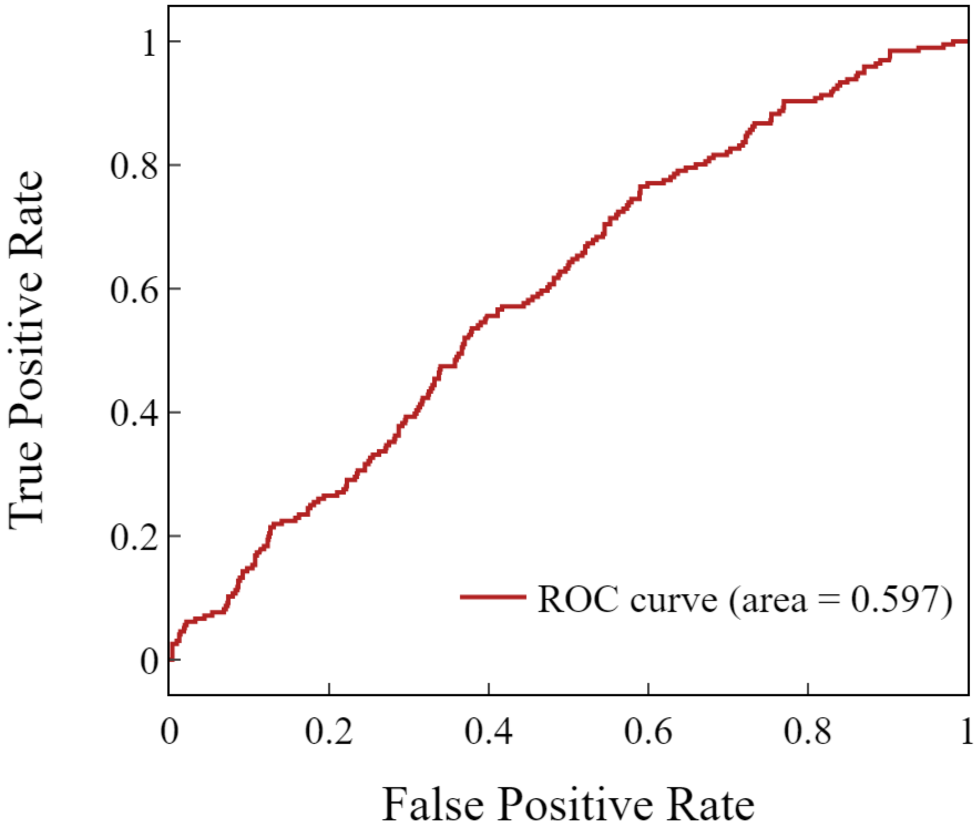

Figure S44. The ROC curve of 6th and 7th PCA components.

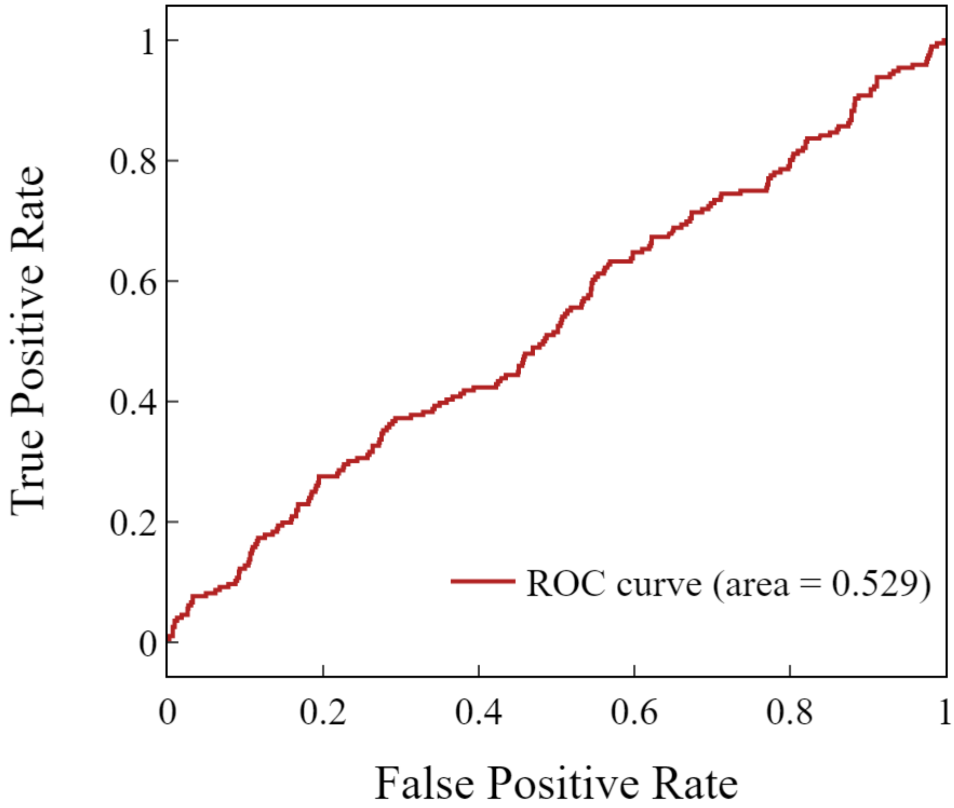

Figure S45. The ROC curve of 6th and 8th PCA components.

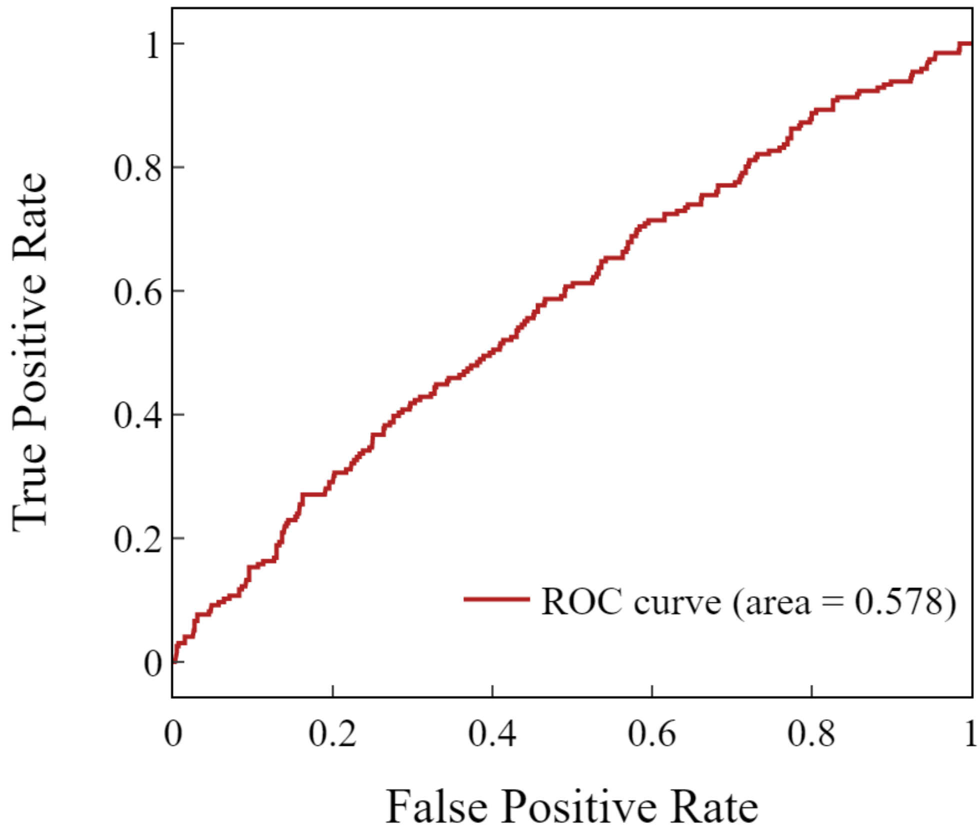

Figure S46. The ROC curve of 6th and 9th PCA components.

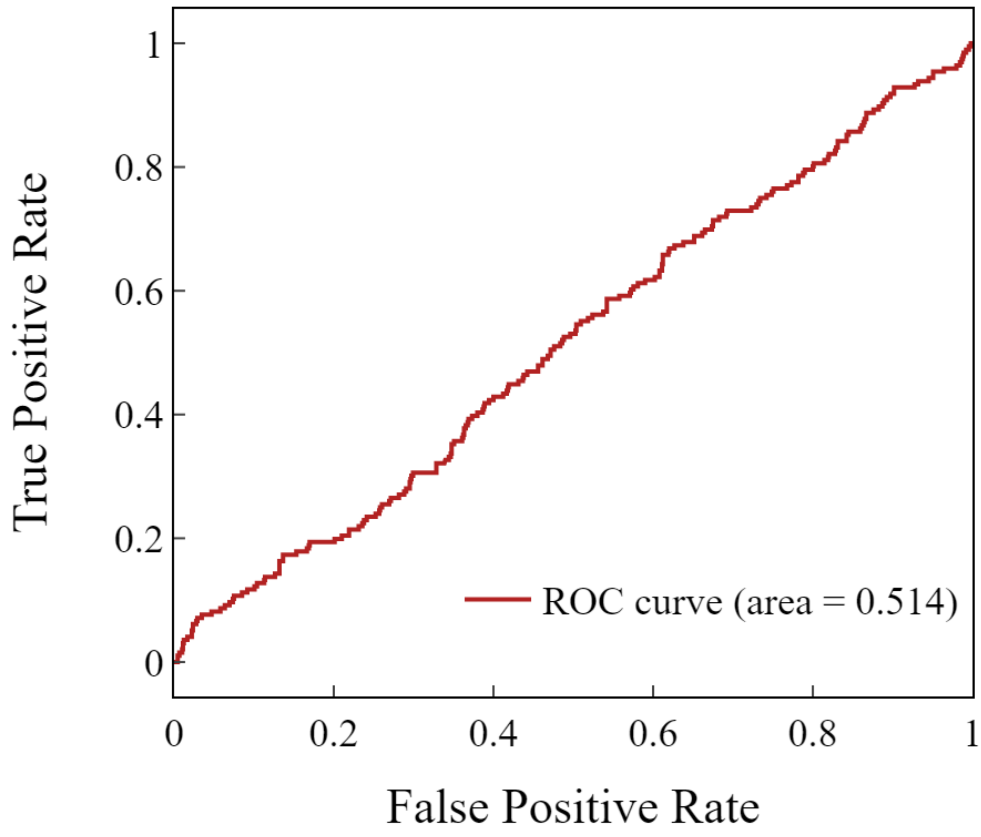

Figure S47. The ROC curve of 6th and 10th PCA components.

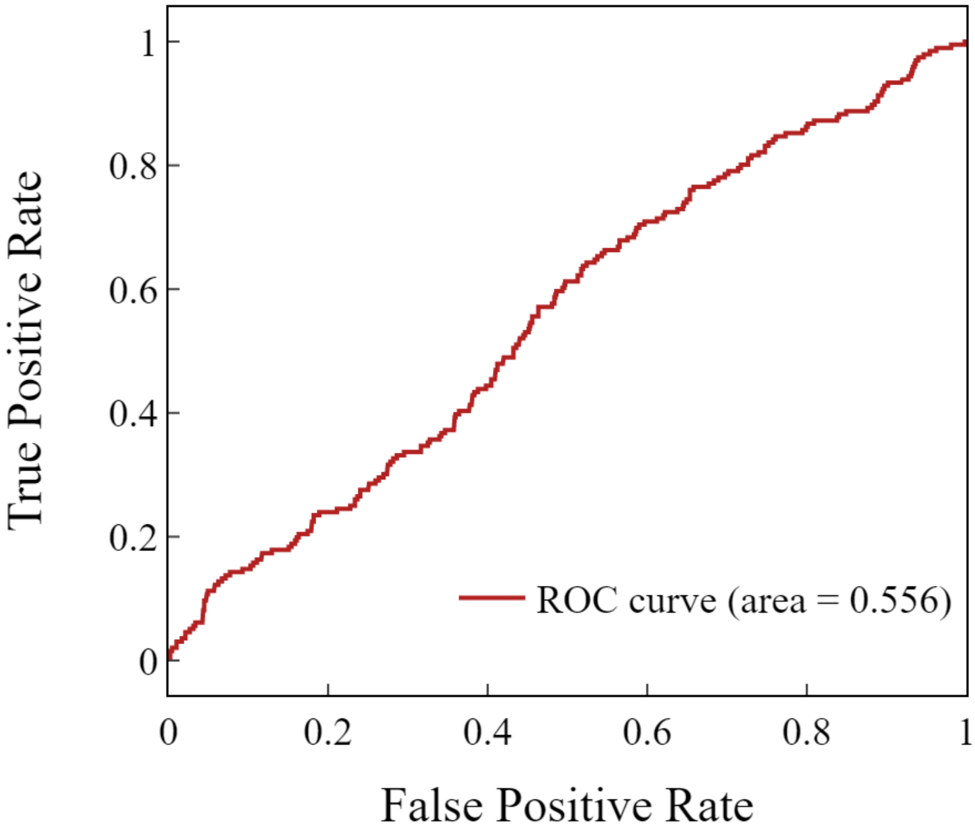

Figure S48. The ROC curve of 6th and 11th PCA components.

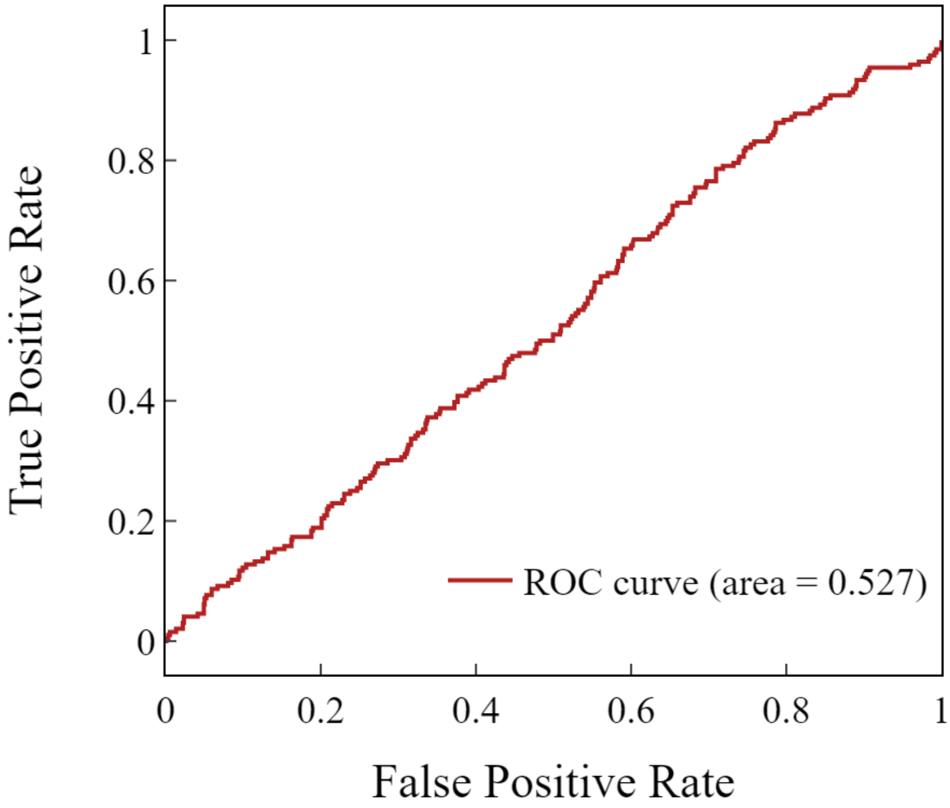

Figure S49. The ROC curve of 7th and 8th PCA components.

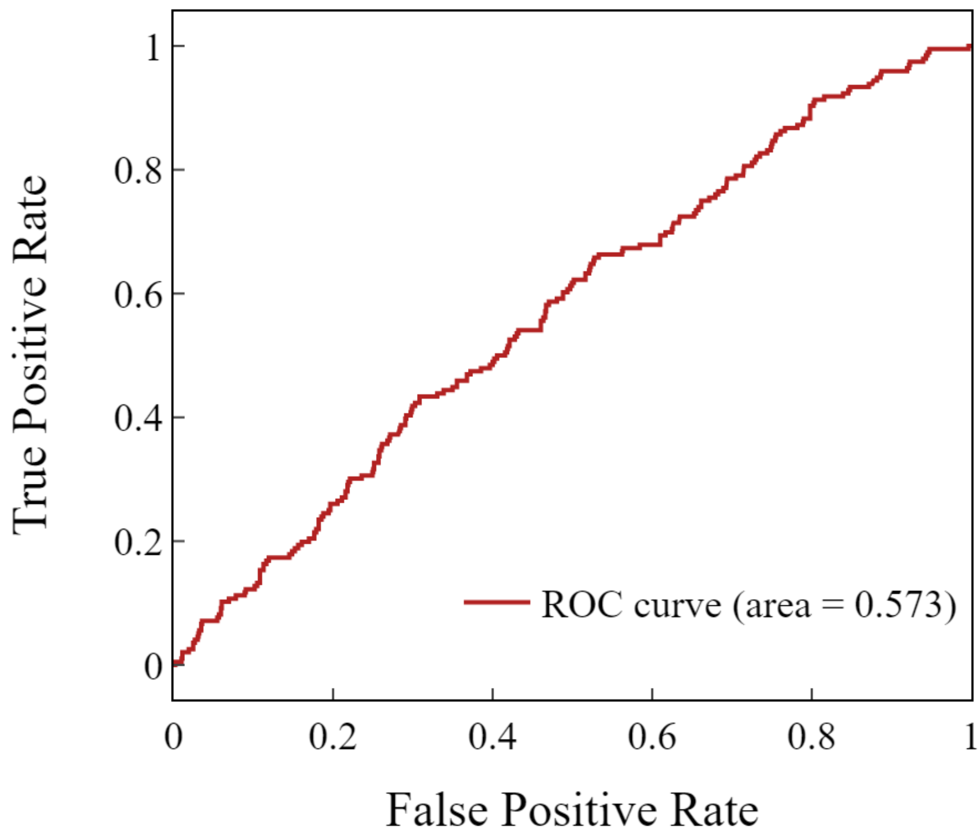

Figure S50. The ROC curve of 7th and 9th PCA components.

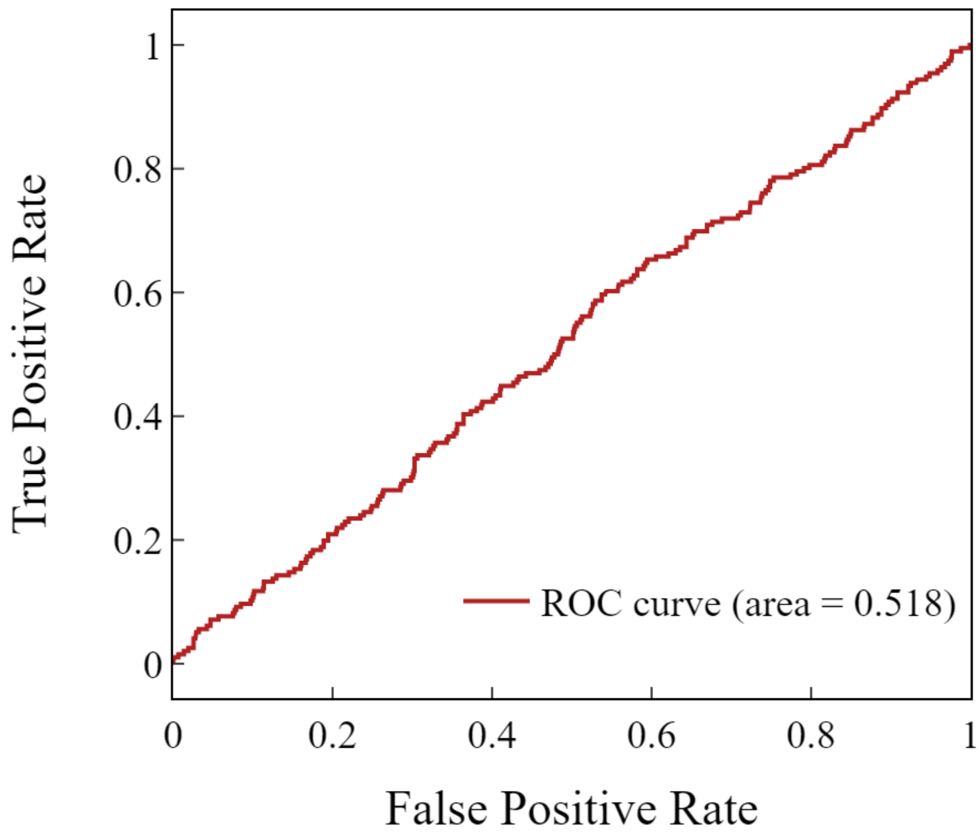

Figure S51. The ROC curve of 7th and 10th PCA components.

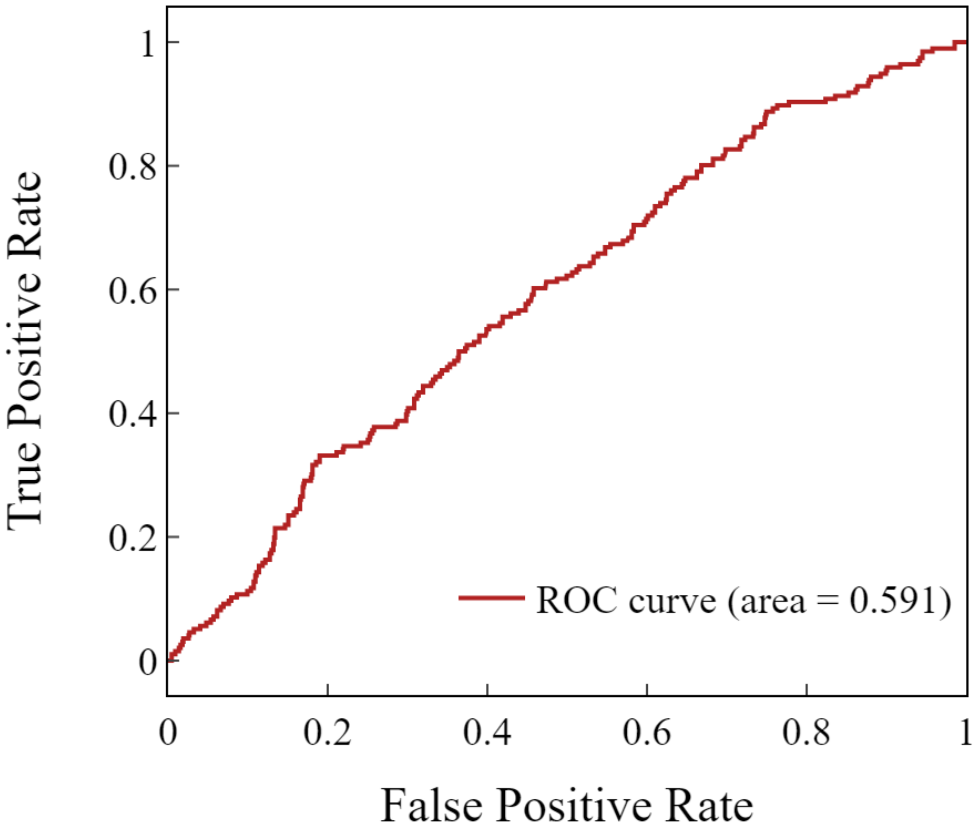

Figure S52. The ROC curve of 7th and 11th PCA components.

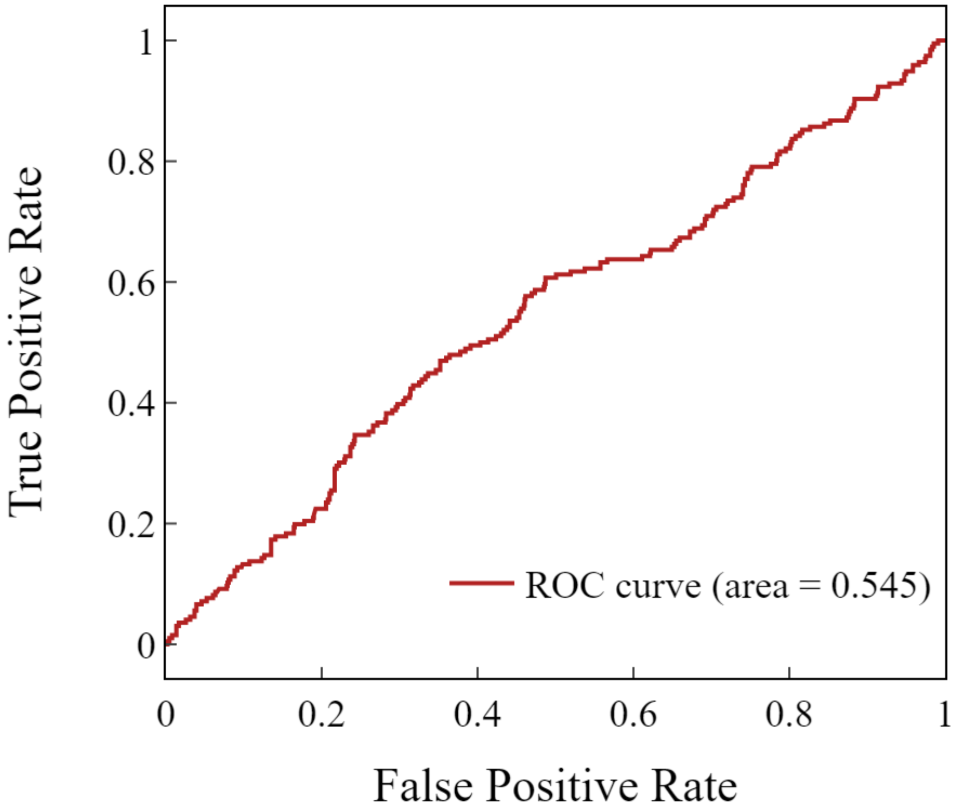

Figure S53. The ROC curve of 8th and 9th PCA components.

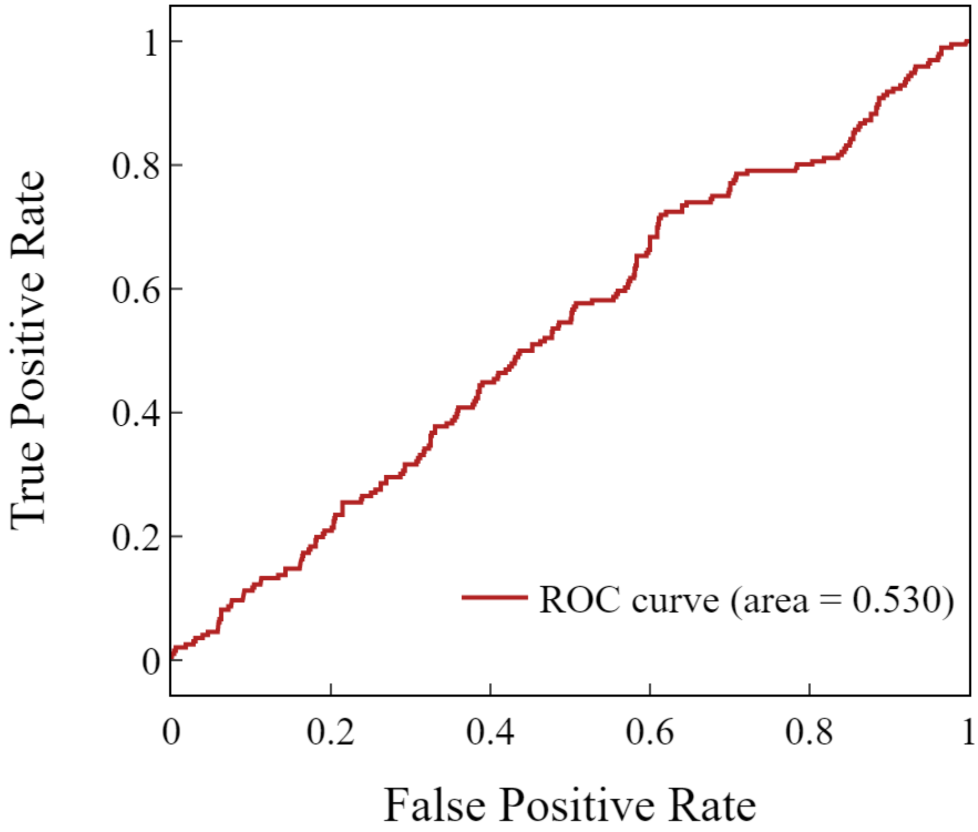

Figure S54. The ROC curve of 8th and 10th PCA components.

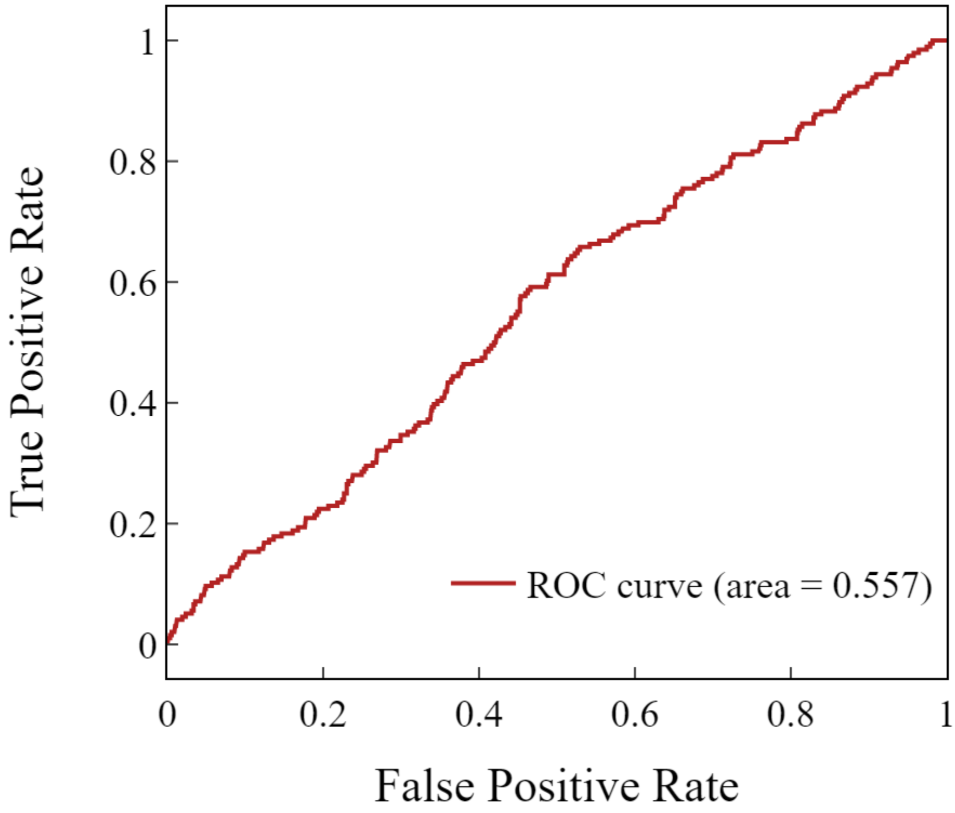

Figure S55. The ROC curve of 8th and 11th PCA components.

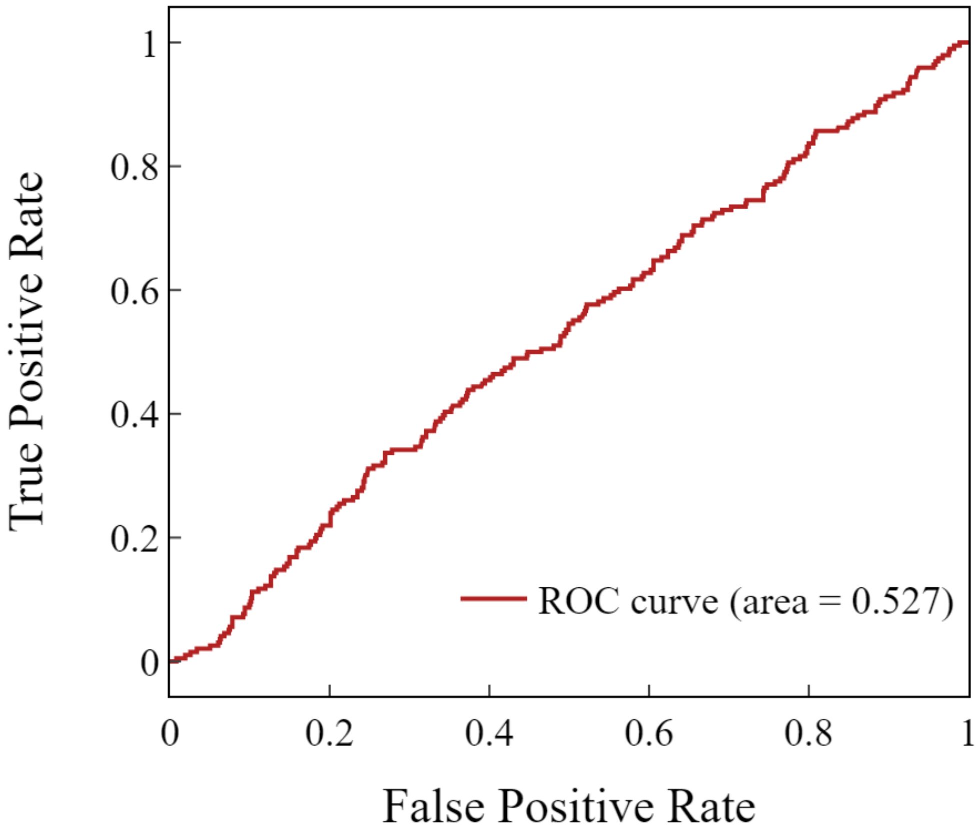

Figure S56. The ROC curve of 9th and 10th PCA components.

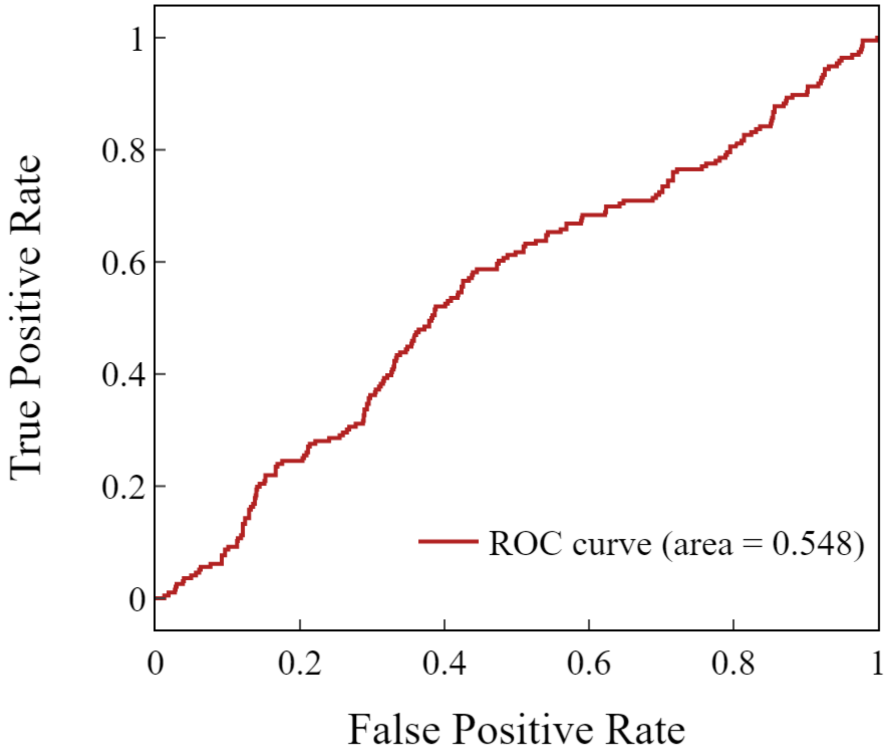

Figure S57. The ROC curve of 9th and 11th PCA components.

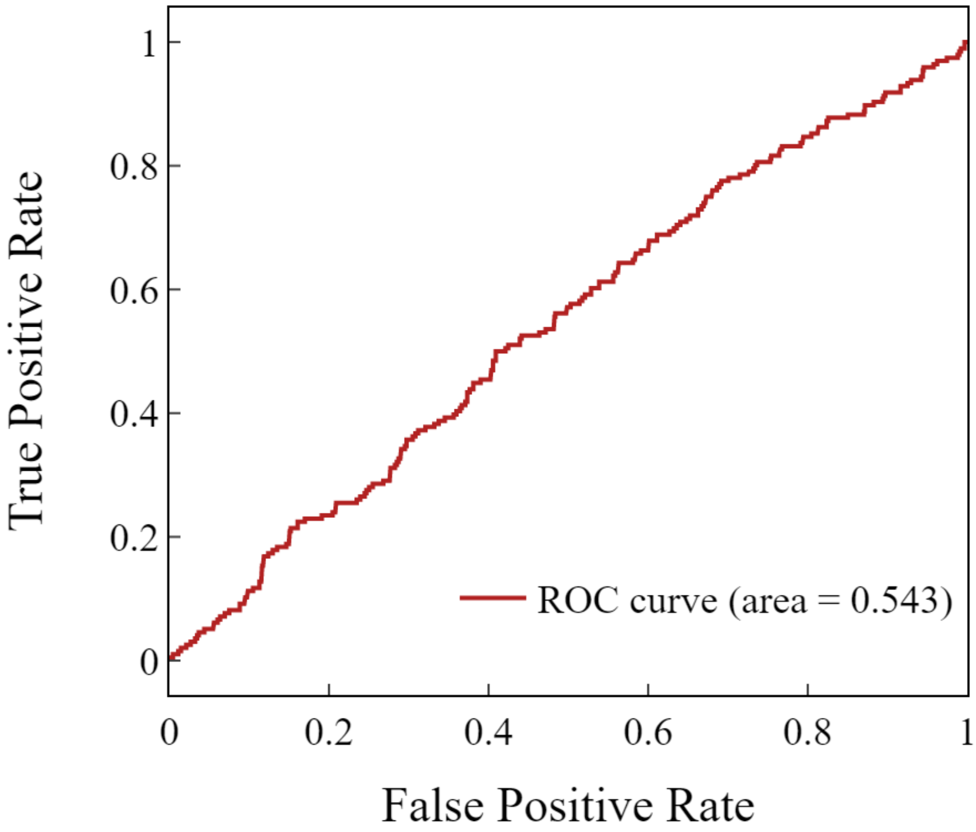

Figure S58. The ROC curve of 10th and 11th PCA components.

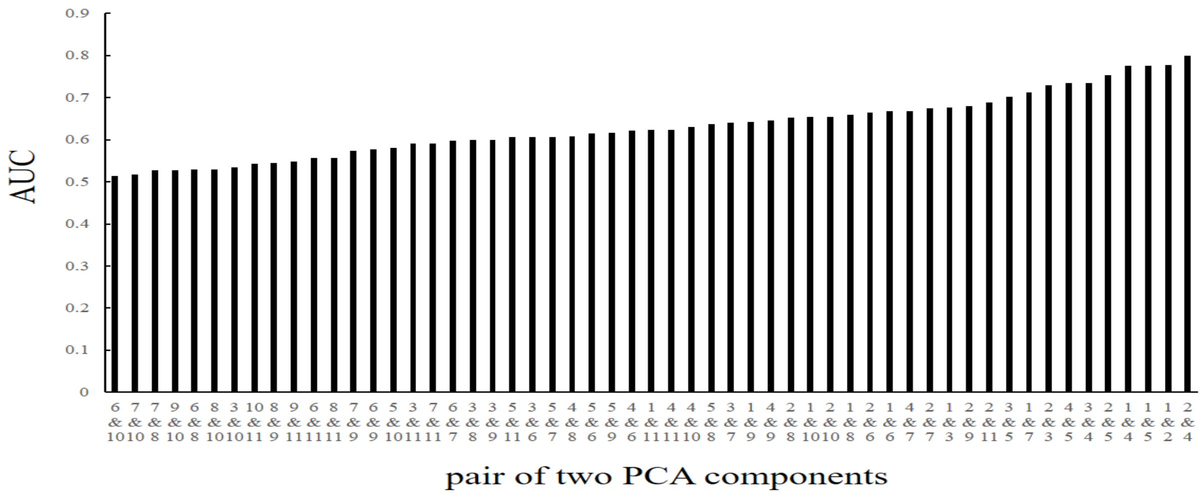

Figure S59. The AUC of ROC curves varied with two PCA component pairs among chosen principal components.

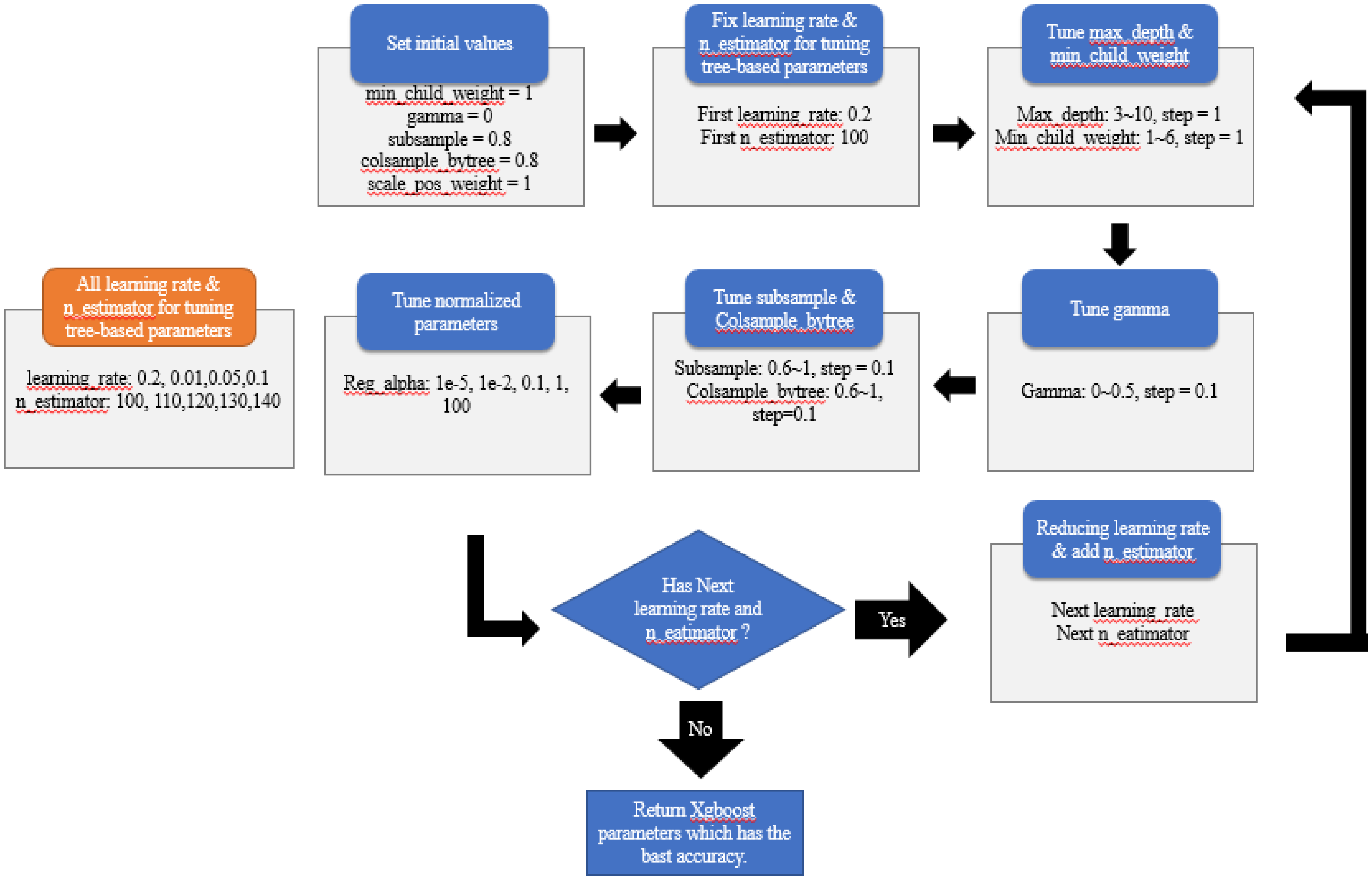

Figure S60. The flow chart and parameters in the algorithm modeling using XGBoost.

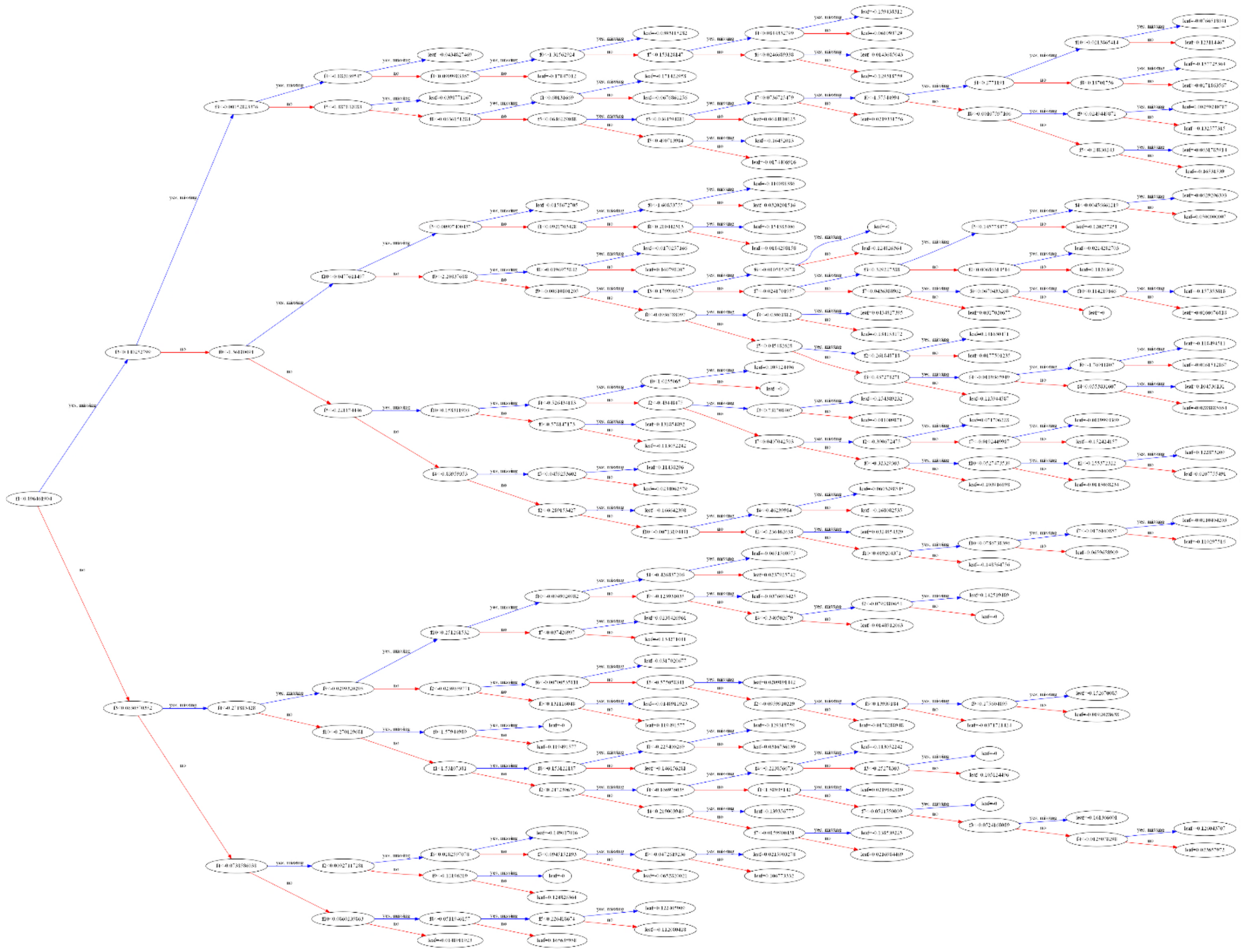

Figure S61. The decision trees in the algorithm modeling using XGBoost.
